# Non-peptidyl small molecule, adenosine, 5′-Se-methyl-5′-seleno-, 2′,3′-diacetate, activates insulin receptor and attenuates hyperglycemia in type 2 diabetic *Lepr*^*db/db*^ mice

**DOI:** 10.1007/s00018-019-03249-4

**Published:** 2019-08-05

**Authors:** Zi-Jian Lan, Zhenmin Lei, Alexandros Yiannikouris, Thirupathi Reddy Yerramreddy, Xian Li, Hayley Kincaid, Katie Eastridge, Hannah Gadberry, Chloe Power, Rijin Xiao, Lei Lei, Olivia Seale, Karl Dawson, Ronan Power

**Affiliations:** 1 Division of Life Sciences, Alltech, Inc, 3031 Catnip Hill Road, Nicholasville, KY 40356 USA; 2 Department of OB/GYN, University of Louisville School of Medicine, MDR Building/Room 121, 511 South Floyd St., Louisville, KY 40202 USA; 3 Chemistry Department, Alltech, Inc, Nicholasville, KY 40356 USA

**Keywords:** Glucose intolerance, Gluconeogenesis, PDK1/AKT/AS160/FOXO1 phosphorylation, In vitro phosphorylation, AML-12, C2C12 cells

## Abstract

The pathophysiology of type 2 diabetes mellitus (T2D) is characterized by reduced or absent insulin receptor (INSR) responsiveness to its ligand, elevated hepatic glucose output and impaired glucose uptake in peripheral tissues, particularly skeletal muscle. Treatments to reduce hyperglycemia and reestablish normal insulin signaling are much sought after. Any agent which could be orally administered to restore INSR function, in an insulin-independent manner, would have major implications for the management of this global disease. We have discovered a non-peptidyl small molecule, adenosine, 5′-Se-methyl-5′-seleno-, 2′,3′-diacetate [referred to as non-peptidyl compound #43 (NPC43)], which restores INSR signaling in the complete absence of insulin. Initial screening of numerous compounds in human HepG2 liver cells revealed that NPC43 significantly inhibited glucose production. The compound was potently anti-hyperglycemic and anti-hyperinsulinemic in vivo, in insulin-resistant T2D *Lepr*^*db/db*^ mice, following either acute or chronic treatment by oral gavage and intraperitoneal injection, respectively. The compound acted at the level of INSR and activated it in both liver and skeletal muscle of *Lepr*^*db/db*^ mice. In cell culture, the compound activated INSR in both liver and skeletal muscle cells; furthermore, it cooperated with insulin to depress *glucose*-*6*-*phosphatase catalytic subunit* (*G6pc*) expression and stimulate glucose uptake, respectively. Our results indicated that the compound directly interacted with INSRα, triggering appropriate phosphorylation and activation of the receptor and its downstream targets. Unlike insulin, NPC43 did not activate insulin-like growth factor 1 receptor in either liver or skeletal muscle. We believe this compound represents a potential oral and/or injectable insulin replacement therapy for diabetes and diseases associated with insulin resistance.

**Electronic supplementary material:**

The online version of this article (10.1007/s00018-019-03249-4) contains supplementary material, which is available to authorized users.

## Introduction

Insulin receptor (INSR) signaling is essential for glucose homeostasis and is initiated by the binding of the extracellular domain of INSR (INSRα) to its natural ligand insulin [1, 2]. Shortly after insulin binding, INSR undergoes conformational changes, resulting in auto-phosphorylation of tyrosine residues at 1146, 1150 and 1151 in the intracellular beta subunits of INSR (INSRβ) to activate the intrinsic tyrosine kinase in INSRβ [3, 4]. Once INSRβ is activated, it triggers phosphorylation of downstream signaling molecules in insulin-sensitive tissues, thus controlling glucose production and uptake [1, 5].

Diabetes is reaching pandemic proportions and approximately 90% of all diabetic cases are type 2 diabetes (T2D) [6, 7]. In T2D, INSR becomes resistant to insulin cues for the activation of downstream signaling which controls hepatic glucose production (gluconeogenesis) and blood glucose clearance (primarily into skeletal muscle); this results in hyperglycemia and glucose intolerance [1, 8]. Finding a small molecule which can selectively activate INSR signaling in an insulin-independent manner represents a novel treatment for diabetes, including T2D.

To date, just a few insulin mimetics have been reported [9–16]. These are predominantly based upon quinone-, polyphenol- or chaetochromin-containing compounds, respectively [9–16]. They were not further developed for several reasons, including: cell toxicity [11], low receptor specificity [15–17], undesirable binding to insulin-like growth factor 1 receptor (IGF1R) [14] and activation of IGF1R [15–17], as well as the inhibition of protein tyrosine phosphatase 1B (PTP1B) [12]. As such the search for novel, non-toxic insulin mimetics which selectively activate INSR continues and is becoming increasingly urgent as insulin costs continue to rise beyond the realm of affordability for many diabetics [18–20] (http://www.cbsnews.com/news/the-rising-cost-of-insulin-horror-stories-every-day/, http://www.nytimes.com/2018/06/22/well/diabetes-patients-at-risk-from-rising-insulin-prices.html).

Here we report the discovery of one such compound which appears to avoid the above pitfalls reported for other candidate INSR agonists: adenosine, 5′-Se-methyl-5′-seleno-, 2′,3′-diacetate [non-peptidyl compound #43 (NPC43)]. It displays low toxicity, retains activity over a broad dose range in vitro and in vivo, selectively activates INSR without affecting related receptor tyrosine kinases or PTP1B. In addition, it is effective both as an oral preparation and injectable and thus may represent a significant advance in the treatment of diabetes mellitus.

## Materials and methods

### Compounds and cell lines

**Fig. 1:**
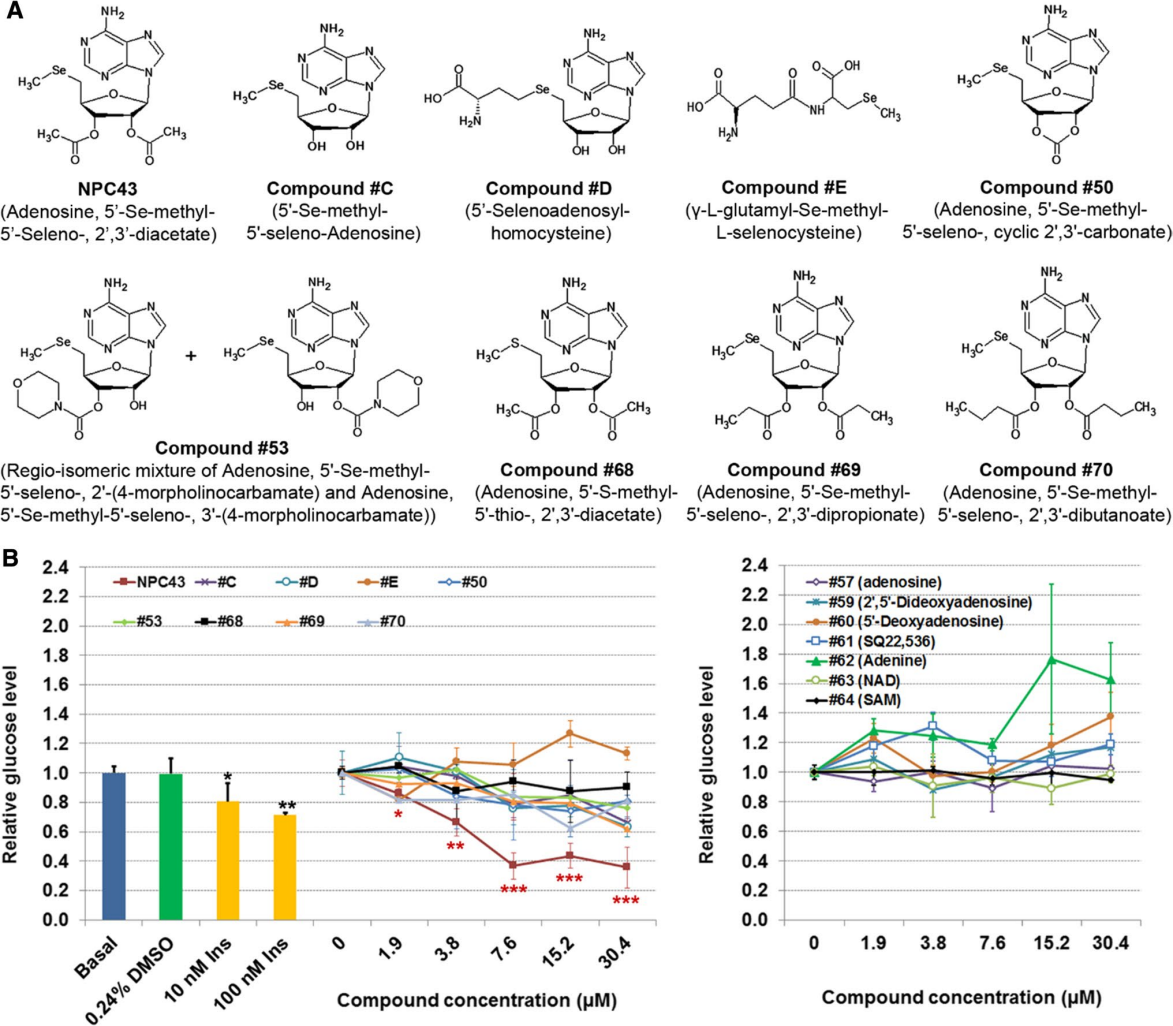
Identification of NPC43 as an inhibitor of glucose production in HepG2 cells. **a** Chemical structures of NPC43 and eight other related compounds. **b** Effects of NPC43 and other listed compounds on glucose production in HepG2 cells. Equal amounts of HepG2 cells were seeded on 96-well plates and cultured in 10% FBS DMEM media overnight. These cells were treated without (basal) or with 0.24% (v/v) DMSO (the maximum volume of tested compound solvent; zero concentration group), insulin or various concentrations of the above listed compounds (left panel) and other related compounds (right panel) from Sigma (Online Resource 2) in serum-free glucose production media for 48 h and then subjected to glucose production assay and cell viability analysis. Relative glucose levels were obtained by normalizing the glucose concentration in culture media by cell number in each sample. Data are presented as mean ± SD for four replicates per group. *P < 0.05, **P < 0.01, ***P < 0.001 vs. 0.24% DMSO group (Student’s *t* test)

NPC43 and Compound #50, #53, #68, #69 and #70 (Fig. 1a) were synthesized as described in the Supplementary Material (Online Resource 1). Compound #C, #D and #E (Fig. 1a) were also synthesized in house (2017, US Patent 9,642,874) and the details of their synthesis are described elsewhere. All other compounds were purchased from Sigma (Online Resource 2). Human hepatoma HepG2, rat hepatoma H4IIE, mouse liver AML-12 and mouse myoblast C2C12 cells were purchased from ATCC (Manassas, Virginia). HepG2 and H4IIE cells were maintained in Eagle’s Minimum Essential Medium (EMEM) supplemented with 10% fetal bovine serum (FBS). AML-12 cells were amplified in Dulbecco’s modified Eagle’s medium (DMEM)/Ham’s F12 (DMEM/F12) media supplemented with 10% FBS, 40 ng/ml dexamethasone (Dex, Sigma) and 1 × ITS solution (containing 0.01 mg/ml bovine insulin, 0.0055 mg/ml human transferrin, 5 ng/ml sodium selenite, Sigma). Mouse C2C12 cells were amplified in DMEM media supplemented with 10% FBS.

### Glucose production in HepG2 cells and cell viability analysis

Equal numbers of HepG2 cells (1.25 × 10^5^ cells/well) were seeded on 96-well plates, and treated without (basal) or with various concentrations of compounds, insulin (Sigma), or 0.24% (v/v) DMSO (the maximum volume of tested compound solvent) in 100 µl of glucose production media (glucose-free, phenol red-free DMEM media supplemented with 20 mM sodium lactate, 2 mM sodium pyruvate and 5 mM HEPES) at 37 °C for 48 h. Glucose levels in the media were determined using Molecular Probes Amplex Red glucose assay kits (Themo-Fisher Scientific). Cell numbers in the culture plate after the above treatments were determined using Promega’s CellTiter96^®^ AQueous One Solution Cell Proliferation Assay kits. Glucose production in the cultured cells was determined by normalizing the glucose concentration in culture media by cell number in each well.

Cell viability analysis was also performed on rat liver H4IIE cells and mouse liver AML-12 cells using Promega’s CellTiter96^®^ AQueous One Solution Cell Proliferation Assay kits, according to the manufacturer’s protocol and instructions. In brief, equal numbers of H4IIE cells (1.1 × 10^5^ cells/well) were seeded on 96-well plates in 10% FBS-containing EMEM for 24 h. Cells were then washed twice with PBS and treated with NPC43 solvent (0.24% v/v DMSO, referred to as zero control) or NPC43 (1.9–30.4 μM) in 100 µl of phenol red-free DMEM media at 37 °C for 24 h. Similarly, AML-12 cells (4 × 10^4^ cells/96-well) were also seeded in 96-well plates and cultured in FBS-, ITS- and Dex-containing DMEM/F12 media overnight. These AML-12 Cells were then washed twice with PBS and treated with NPC43 solvent (0.24% v/v DMSO, referred to as zero control) or NPC43 (1.9, 3.8 and 7.6 μM) in 100 µl of phenol red-free DMEM media at 37 °C for 24 h. After the above treatments, H4IIE and AML-12 cells were incubated with Promega’s CellTiter96^®^ AQueous One Solution and their absorbance at OD490 nm (representing cell viability of cultured cells) was measured using a microplate reader. Four replicates were performed for each group in the above experiments. Student’s *t* test analysis was performed to determine the *P* value between the zero control group and the NPC43-treated group.

### Animals, acute and chronic intraperitoneal (i.p.) and per os (p.o.) treatment and blood glucose assay

Male *Lepr*^*db/db*^ mice (C57BL/6J strain) and wild-type C57 mice were purchased from The Jackson Laboratory (Bar Harbor, Maine), and housed in a pathogen-free vivarium with free access to chow and water. All animal studies were pre-approved by Alltech, Inc. and performed according to the US National Institute of Health’s Animal Welfare guidelines. Overnight- and 2 h-fasted male 8- to 10-week-old *Lepr*^*db/db*^ mice were subjected to acute i.p. and p.o. (by oral gavage) treatment with NPC43 or its vehicle [0.2% (v/v) DMSO/saline for i.p. injection or 0.5% (w/v) carboxymethylcellulose (CMC) for oral gavage], respectively. Following the treatments, mice were returned to their cages with free access to water but not chow for 1, 2, 3, 4, 5 or 8 h. Blood glucose levels in each mouse were determined using a glucometer with a maximum ceiling reading of 600 mg/dl. Blood glucose levels over 600 mg/dl were counted as 600 mg/dl in the data analysis. The change in blood glucose level in individual mice was obtained by subtracting the glucose level right before i.p. injection or oral gavage from the blood glucose level at each time-period after i.p. injection or oral gavage. For chronic p.o. treatment with NPC43, 65-day-old male *Lepr*^*db/db*^ mice were fasted for 2 h and blood glucose levels at day 0 were determined using a glucometer. These mice were then fed daily (by oral gavage) with 0.5% (w/v) CMC or NPC43 [1.08 and 5.4 mg/kg body weight (BW)] for 5, 7 and 9 consecutive days. After each oral administration, animals were returned to their cages with free access to water and chow. At 5, 7 and 9 days post oral gavage, *Lepr*^*db/db*^ mice were fasted for 2 h and then blood glucose levels were determined using a glucometer. For chronic i.p. treatment with NPC43, male *Lepr*^*db/db*^ mice at 38 or 41 days of age were injected daily with physiological saline containing 0.2% (v/v) DMSO/saline, or NPC43 (0.136 mg/kg BW) for 43–90 days. Any animal mortality and abnormality in mouse gross morphology and animal behavior in *Lepr*^*db/db*^ mice were recorded daily during the above chronic i.p. treatment time-periods. After chronic i.p. treatments, animals were fasted overnight and then subjected to blood glucose, hemoglobin A1c (HbA1c) and insulin assays, glucose tolerance tests, serum alanine aminotransferase (ALT) analysis or tissue collections.

### Analysis of blood HbA1c, serum glycated-HbA1c, insulin and ALT levels in T2D mice and glucose tolerance test

After daily i.p. treatment with DMSO/saline or NPC43 for 52 days, *Lepr*^*db/db*^ mouse serum was collected and subjected to a glycated-HbA1c assay using Kamiya Biomedical Company’s mouse glycated HbA1c ELISA kit (KT-58225, Seattle, WA). After daily i.p. treatment with DMSO/saline or NPC43 for 90 days, blood samples (collected from the *Lepr*^*db/db*^ mouse tail) were subjected to a HbA1c assay using Crystal Chem’s mouse HbA1c kit (Cat #80310, Downers Grove, IL, USA). Serum glycated-HbA1c or blood HbA1c levels in NPC43-treated mice were normalized to DMSO/saline-treated mice to obtain the relative HbA1c levels. The insulin and ALT levels in mouse sera were determined using Thermo-Fisher Scientific’s Mouse Insulin ELISA kit (Cat# EMINS) and Sigma’s ALT Activity Assay kit (Cat # MAK052), respectively. Glucose tolerance tests were performed on overnight-fasted *Lepr*^*db/db*^ mice and the area under the curve (AUC) in glucose tolerance tests was calculated as described previously [21]. These glucose tolerance tests were repeated three times in *Lepr*^*db/db*^ mice.

### Analysis of *G6pc* RNA expression in HepG2 cells, AML-12 cells and liver tissues from *Lepr*^*db/db*^ mice after NPC43 treatment

For analysis of NPC43 effects on *G6PC* expression, HepG2 cells were treated without or with 100 nM insulin or 7.6 μM NPC43 in serum-free EMEM media for 40 h. For the analysis of *G6pc* expression in mouse liver cells, AML-12 cells were pretreated without or with NPC43 (1.9 or 3.8 μM) in DMEM/F12 media supplemented with 10% FBS but without ITS (insulin, transferrin and sodium selenite) and Dex for 24 h. After 24 h treatment, AML-12 cells were washed twice with PBS and then treated without or with insulin (10 or 100 nM), NPC43 (1.9 or 3.8 μM) or both in the serum-free DMEM/F12 media for 6 h. For RNA analysis of *G6pc* expression in diabetic mice, liver tissues from male *Lepr*^*db/db*^ mice following chronic p.o. treatment with 0.5% (w/v) CMC or NPC43 (1.08 and 5.4 mg/kg BW, p.o. daily for 9 days) or chronic i.p. injection with 0.2% (v/v) DMSO/saline or NPC43 (0.136 mg/kg BW, daily for 52 days) were collected. Total RNA from the above cultured liver cells or *Lepr*^*db/db*^ mouse liver tissues was isolated using a Qiagen RNAeasy RNA isolation kit according to the Manufacturer’s protocol. These RNA samples were subjected to real-time PCR (QRT-PCR) analysis using Applied-Bioscience’s RT kit and predesigned Taqman probes (*G6PC*, Hs00609178_m1; *G6pc*, Mm00839363_m1; *ACTB*, Hs01060665_g1; *Actb*, Mm02619580_g1, Cat #4331182, ThermoFisher Scientific), as described previously [22].

### Protein sample preparation, Western blot analysis, ELISA and immunoprecipitation

HepG2 cells were serum-starved overnight and then treated without [before NPC43 treatment, referred to as 0 min (min)] or with NPC43 (7.6 μM) for 30, 60 or 90 min. Mouse C2C12 cells were differentiated using 0.5% horse serum-containing DMEM media (differentiation media) for 7 days, as previously described [23]. These differentiated C2C12 cells were serum-starved overnight and then treated without (control) or with insulin (0.2 μM), NPC43 (7.6 μM), or both in serum-free DMEM media at 37 °C for 5 min, 60 min or 6 h. For protein analysis of Insr signaling or Igf1r in the liver and gastrocnemius skeletal muscle, male *Lepr*^*db/db*^ mice at postnatal day 38 were i.p. injected daily with 0.2% (v/v) DMSO/saline or NPC43 (0.136 mg/kg BW) for 52 days. Protein samples from the above treated human or mouse cells were collected using a RIPA buffer containing complete proteinase and phosphatase inhibitors (Thermo-Fisher Scientific, Waltham, MA, USA). Liver and skeletal muscle protein samples from DMSO/saline- or NPC43-treated *Lepr*^*db/db*^ mice were prepared as described previously [24, 25]. Protein levels in these samples were determined using a Micro-BCA protein assay kit (Thermo Scientific-Piece Biotechnology, Rockford, IL, USA).

Western blot analyses were performed using 5 μg protein extracts from HepG2 cells, 8 μg from differentiated C2C12 cells or 100 μg liver or skeletal muscle proteins from *Lepr*^*db/db*^ mice, as described previously [25]. Primary antibodies against pINSRβ at Y1146 (Cat #3021) or Y1150/1151 (Cat #3024), INSRβ (Cat #3025), pPDK1 at S241 (Cat #3438), PDK1 (Cat #3062), pAKT at T308 (Cat #2965) or S473 (Cat #4060), AKT (Cat #4691), pFOXO1 at T24 (Cat #9464) or S256 (Cat #9461), FOXO1 (Cat #2880), pAS160 at S588 (Cat #8730), AS160 (Cat #2670), pIGF1Rβ at Y1131 (Cat #3021), IGF1Rβ (Cat #3027), ACTB (Cat #8457) and β-tubulin (Cat #2146) were purchased from Cell Signaling Technology (Danvers, MA, USA). Antibodies against Gapdh were obtained from Abcam (Cat#ab181602, Cambridge, MA, USA). Protein band densities in the Western blots were determined using the NIH ImageJ software (Rasband, W.S., ImageJ, U. S. National Institutes of Health, Bethesda, Maryland, USA,1997–2018, https://imagej.nih.gov/ij/) and then normalized by Gapdh, β-tubulin or Actb/ACTB level in each sample. In addition, the ratio of phosphorylated form to total protein level of the respective insulin signaling molecules was also calculated.

ELISA assays were performed to determine the levels of pInsrβ Y1146 and pInsrβ Y1150/1151 in four- and six-hundred micrograms of liver protein extracts, respectively, from 0.2% (v/v) DMSO/saline- or NPC43 (0.136 mg/kg BW)-treated *Lepr*^*db/db*^ mice (i.p. daily for 52 days) using the PathScan Phospho-Insulin Receptor β (Tyr1146 or Tyr1150/1151) Sandwich ELISA kits (Cell Signaling Technology, Danvers, MA, USA). The protein levels of pIGF1Rβ Y1131 in liver (50 μg total protein) and gastrocnemius skeletal muscle (150 μg total protein) of 0.2% (v/v) DMSO/saline- or NPC43 (0.136 mg/kg BW)-treated *Lepr*^*db/db*^ mice (i.p. daily for 52 days) were also determined using the PathScan Phospho-IGF-1 Receptor β (Tyr 1131) Sandwich ELISA kit (Cell Signaling Technology).

In addition, immunoprecipitation (using the Themo-Fisher’s Dynabead™ Protein G-immunoprecipitation kit) followed by Western blot analysis was performed to determine pAS160 protein levels in the liver (using 1 mg total protein) and skeletal muscle (using 400 μg total protein) of 0.2% DMSO/saline- or NPC43 (0.136 mg/kg BW)-treated *Lepr*^*db/db*^ mice (i.p. daily for 52 days). Protein levels of pAS160 in each sample were normalized to the corresponding total AS160 protein levels. Also, six hundred micrograms of liver and skeletal muscle protein extracts from each DMSO/saline- or NPC43-treated *Lepr*^*db/db*^ mouse (0.2% DMSO/saline or 0.136 mg/kg BW NPC43, i.p daily for 52 days) were incubated with Igf1r antibodies (Cell Signaling Technology, Cat# 3027, 1:100 dilution) at 4 °C overnight to pull down Igf1r proteins using the Dynabead™ Protein G-immunoprecipitation kit (Thermo-Fisher). Phosphorylated Igf1rβ at Y1131 and total Igf1rβ protein levels in these immunoprecipitated samples were detected by Western blot analysis using antibodies specific for each (Cell Signaling Technology, Cat #3021 and #3027).

### Glucose uptake assay in mouse AML-12 and differentiated C2C12 cells

Equal numbers of AML-12 cells were seeded on 96-well plates (1.5 × 10^5^/well) and cultured in 10% FBS but ITS/Dex-free DMEM/F12 media overnight. Then, these cells were serum-starved overnight and treated without (basal) or with insulin (10 and 100 nM), NPC43 (1.9, 3.8 and 7.6 μM) or both insulin (10 nM) and NPC43 in glucose- and phenol red-free DMEM media at 37 °C for 1.5 h. Similarly, equal numbers of C2C12 cells were seeded on 96-well plates (5000 cells/well) and cultured in 10% FBS DMEM media (with daily replacement of media) at 37 °C for 5 days. At day 5 of culture, C2C12 cells were differentiated using 0.5% horse serum-containing DMEM media for 7 days, as previously described [23]. At day 7 post-differentiation, differentiated C2C12 cells were rinsed with PBS twice and pretreated without or with NPC43 (3.8 and 7.6 μM) in serum- and glucose-free DMEM media overnight. Then these C2C12 cells were treated without (basal) or with insulin (0.2 μM), NPC43 (3.8 and 7.6 μM), or both insulin and NPC43 in serum-, glucose- and phenol red-free DMEM media at 37 °C for 1.5 h. Following treatment, AML-12 or differentiated C2C12 cells were incubated with 1 mM 2-deoxyglucose (2DG) at room temperature for 30 min. These 2DG-treated cells were then subjected to glucose uptake analysis using Promega’s Glucose Uptake-Glo Assay kit, according to the manufacturer’s protocol. Luminescence intensities (RLU) in the samples were determined using a 0.5-s integration on the Bio-Tek luminometer. Experiments were repeated twice.

### In vitro PTP1B and receptor tyrosine kinase assays

Studies of NPC43 effects on the activities of PTP1B were performed using Abcam’s PTP1B inhibitor Screening Assay kit (Cambridge, MA, USA), according to the manufacturer’s procedures. In brief, NPC43 (0.5–100 μM), suramin (a positive PTP1B inhibitor, 10 μM) or 0.79% (v/v) DMSO (the NPC43 solvent) were incubated with 2.5 ng recombinant PTP1B and 75 μM PTP1B substrate (INSRβ amino acid residues 1142–1153, pY-1146) at 30 °C for 30 min. Abcam’s Red Assay Reagents were then added to tested samples to determine the released phosphate concentration from the phosphorylated INSRβ substrate. Triplicates were examined in each treatment group.

Effects of NPC43 on the tyrosine kinase activities of active INSRβ (1101–1382 aa residue), IGF1Rβ, HER4, KDR, PDGFRα and PDGFRβ were examined using Promega’s Kinase Selectivity Profiling System (Madison, WI, USA), according to the manufacturer’s protocol. NPC43 solvent (5% DMSO in PBS buffer) was also included as a positive control (referred to 100% kinase activity) for these kinase assays.

### In vitro binding of NPC43 with Insrα from rat liver Insr protein

Specific antibodies against Insrα (Abcam, Cat #36550) were linked to Protein G-linked Dynabeads (Thermofisher) to obtain Insrα-antibody-conjugated Dynabeads. NPC43 (3.8 μM) was incubated without or with 81.5 or 326 nM native Insr proteins purified from rat liver (Sigma, diluted in PBS) at 4 °C overnight and then subjected to immunoprecipitation analysis using the above Insrα-antibody-conjugated Dynabeads (Insrα-pAb beads). The same amounts of Insrα-pAb beads were used in each reaction to remove Insrα-bound NPC43. Non-antibody-conjugated Dynabeads (Non-pAb beads) were also incubated with 3.8 μM NPC43 as a Control. The concentration of NPC43 in the supernatant (after removal of Insrα-bound NPC43 by Insrα-pAb beads or non-specific bound NPC43 by Non-pAb beads or Insrα-pAb beads) in each sample was determined by mass spectrometry analysis using a UPLC-ESI-VionIMS-QTOF instrument (Waters Corp., Milford, MA, USA), equipped with a RP-C8 analytical column (2.1 mm × 100 mm, Waters Corp). Details of quantitative determination of NPC43 concentration in the tested samples by mass spectrometry analysis are described in the Online Resource 3.

### Tyrosine (Tyr)/phenylalanine (Phe) and tryptophan (Trp) fluorescence analysis of recombinant INSRα protein after incubation with NPC43

Tyr/Phe and Trp fluorescence signals in recombinant INSRα proteins (0.25 μM, aa 1–956, Cat # 11081-H08H, ThemoFisher) after incubation with 0.006% DMSO, 1 and 4 μM NPC43 (in PBS buffer solution) were determined by previously described methods [12, 26]. The fluorescence spectra of NPC43 at concentrations of 1 and 4 μM in the absence of recombinant INSRα protein were also collected to determine the potential intrinsic fluorescence of NPC43. Experiments were repeated three times.

### In vitro phosphorylation of rat liver Insr protein or recombinant IGF1R by NPC43

In vitro phosphorylation of native Insr protein (containing both alpha and beta subunits) from rat liver was performed according to Sigma’s protocol with the following modifications: In brief, 10 µl of native insulin receptor solution [containing 0.8 µl of 6.52 μM original native Insr stock solution (Sigma, Catalog #I9266; diluted in enzyme dilution buffer containing 50 mM HEPES, pH 7.6, 150 mM NaCl and 0.1% Triton X-100)] was incubated with an equal volume of solutions containing insulin, DMSO or NPC43 (diluted in 50 mM HEPES, pH 7.6 and 100 µg/ml bovine serum albumin) on ice for 30 min. Then 20 µl of 2 × kinase buffer containing 0.2 mM ATP, 50 mM HEPES, pH 7.6, 50 mM MgCl_2_ and 4 mM MnCl_2_ were added to the above reactions, mixed and incubated on ice for 45 min. Then, 5 μl of the above reactions was immediately subjected to Western blot analysis to detect the activated Insr (i.e., pInsrβ at Y1146 and Y1150/1151) using pInsrβY1146, pInsrβY1150/1151 or both antibodies. Protein band density in Western blots was determined using NIH Image J software. Experiments were repeated three times.

Similarly, in vitro phosphorylation of IGF1R proteins was studied using recombinant human IGF1R proteins containing both alpha and beta subunits (400 ng IGF1R protein/reaction, Cat. TP314928, Origene, Rockville, MD, USA). Phosphorylated IGF1Rβ at Y1131 and total IGF1Rβ protein levels in these in vitro phosphorylation reactions were detected by Western blot analysis using their specific antibodies (Cell Signaling Technology, Cat #3021 and #3027). Experiments were repeated three times.

### Statistical Analysis

Where applicable, a Student’s *t* test was used to determine the statistical significance of difference among treatment groups, with a *P* value less than 0.05 being deemed significant.

## Results

### NPC43 inhibited glucose production from human HepG2 cells

Excess hepatic glucose production and hyperglycemia are hallmarks of T2D [8]. Accordingly, the human liver cell line, HepG2, was used as a screening tool for insulin mimetics. Our recent studies demonstrated that a combination of three naturally occurring compounds (Compound #C, #D, and #E; Fig. 1a) displayed glucose-lowering activity in cultured hepatocytes and a T2D mouse model (2017, US Patent 9,642,874). Based on these three core structures, a range of chemical variants (Fig. 1a) were synthesized (Online Resource 1) and a sub-group of six compounds, NPC43, Compound #50, #53, #69, #70 and #68 (Fig. 1a) was found to suppress glucose production from HepG2 Cells (Fig. 1b, left panel).

Of these compounds, NPC43 was the most promising, with the glucose-lowering activity of 3.8 µM NPC43 being equivalent to 100 nM insulin in these cells (Fig. 1b). At higher concentrations, NPC43 was much more potent than 100 nM insulin and reduced glucose production from HepG2 cells by ≥ 40% (Fig. 1b). The replacement of a diacetyl ester at 2′ and 3′ position of NPC43 by hydrogen molecules (Compound #C), cyclic carbonate (Compound #50), morpholino carboxylate (#53), dipropanoyl ester (#69) and dibutanoyl ester (#70) resulted in weaker inhibition of glucose production from HepG2 cells (Fig. 1b, left panel). A slight decrease in glucose production was observed after treatment with Compound #D or Compound #68 (the sulfur analog of NPC43; Fig. 1b). Compound #E did not inhibit glucose production from HepG2 cells (Fig. 1b, left panel), nor did we observe any glucose reduction associated with the following compounds (Online Resource 2): adenosine (Compound #57), 2′,5′-dideoxyadenosine (Compound #59), 5′-deoxyadenosine (Compound #60), SQ22,536 (Compound #61), adenine (Compound #62), β-nicotinamide adenine dinucleotide hydrate (NAD, Compound #63) and *S*-(5′-adenosyl)-l-methionine iodide (SAM, Compound #64) (Fig. 1b, right panel).

### No toxic effects of NPC43 on cultured liver cells and the measurement of ALT levels in the sera of T2D *Lepr*^*db/db*^ mice

The effects of NPC43 on liver cell viability were investigated on several cultured liver cell lines including human HepG2, rat liver H4IIE and mouse liver AML-12 cells. As shown in Online Resource 4 (Panel A, B), treatment with NPC43 at doses up to 30.4 μM did not cause significant changes in cell viability in both HepG2 and H4IIE cells. In mouse AML-12 cells, NPC43 treatment at doses up to 7.6 μM also did not affect cell viability (Panel C, Online Resource 4). These results suggest that NPC43 at the tested doses was not cytotoxic to liver cells.

ALT levels in serum are an accepted biomarker of liver damage. To test whether NPC43 is hepatotoxic in vivo, 38-day-old diabetic *Lepr*^*db/db*^ mice [27] were i.p. treated with 0.2% (v/v) DMSO/physiological saline or NPC43 (0.136 mg/kg BW) daily for 52 days following which serum ALT levels in these DMSO/saline- or NPC43-treated mice, as well as age-matched wild-type C57 mice, were examined. As shown in Online Resource 4 (panel D), serum ALT levels in saline-treated *Lepr*^*db/db*^ mice were significantly higher than non-diabetic C57 mice. However, chronic NPC43 treatment resulted in significantly lower serum ALT levels (relative to the DMSO/Saline-treated animals) in *Lepr*^*db/db*^ mice (Online Resource 4, panel D). These results indicate that chronic treatment with NPC43 at the tested dose may ameliorate diabetic hepatotoxicity in *Lepr*^*db/db*^ mice.

### Acute i.p. and p.o. administration of NPC43 rapidly reduced blood glucose levels in T2D *Lepr*^*db/db*^ mice

**Fig. 2:**
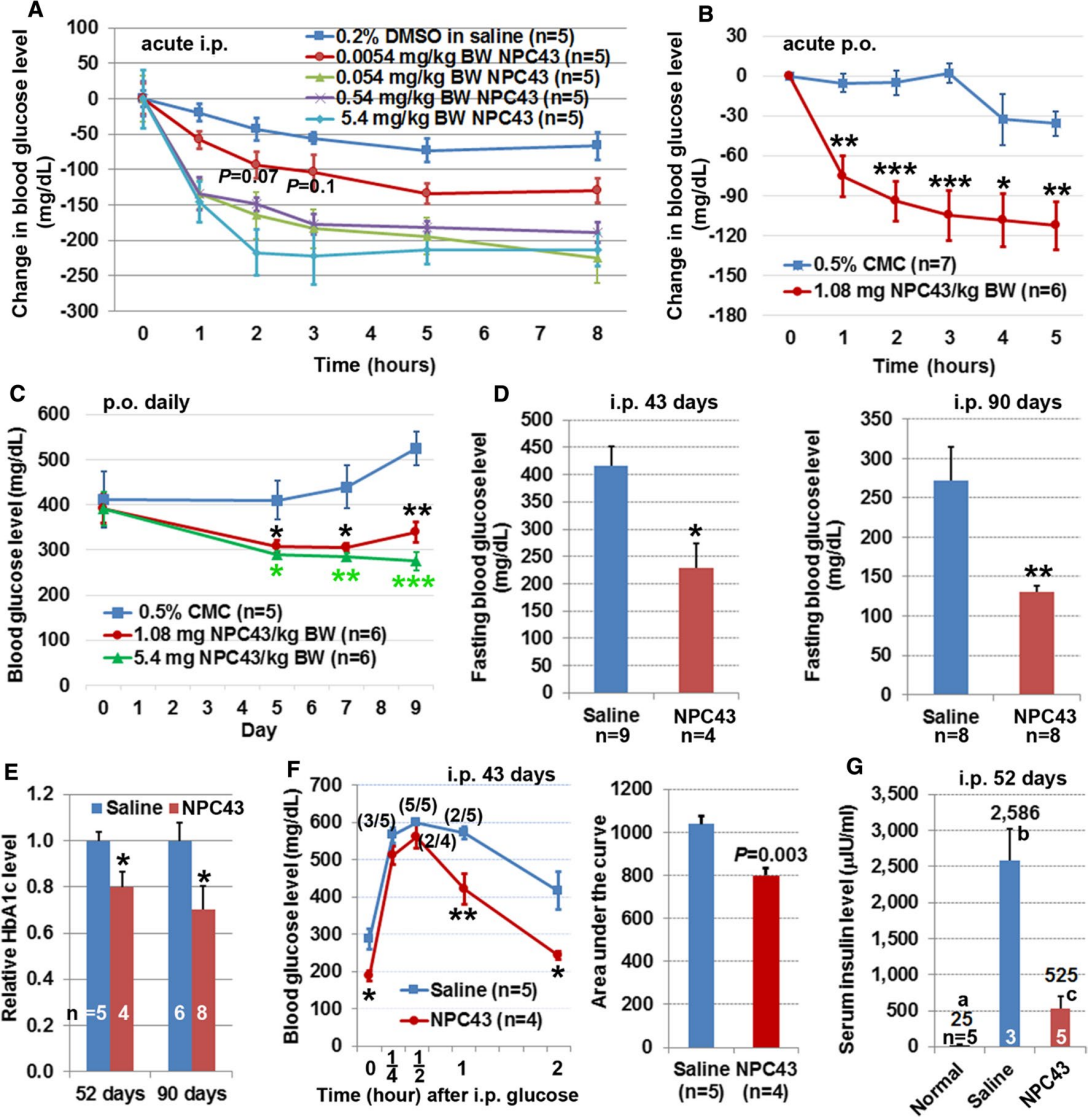
Anti-diabetic activity of NPC43 in *Lepr*^*db/db*^ mice. **a**, **b** Decreased blood glucose levels in *Lepr*^*db/db*^ mice after acute (**a**) i.p. and (**b**) p.o. treatment with NPC43. In **a**, 8- to 10-week-old *Lepr*^*db/db*^ mice were fasted overnight and then i.p. injected with 0.2% (v/v) DMSO/physiological saline or indicated doses of NPC43. In **b**, adult *Lepr*^*db/db*^ mice were fasted for 2 h and then p.o. administered with 0.5% (w/v) carboxymethylcellulose (CMC) or NPC43 (1.08 mg/kg BW). Blood glucose levels in *Lepr*^*db/db*^ mice right before and after i.p. or p.o. treatment at indicated time points (under fasting conditions but having free access to water) were examined. The change in blood glucose level in each mouse was obtained by subtracting the glucose level right before i.p. injection or oral gavage (0 time point) from the blood glucose level at each time-period after i.p. injection or oral gavage. In **a**, with the exception of the *P* values shown (which relate to 2- and 3-h-time points of the 0.0054 mg/kg BW treatment), all other reductions were significant (*P* at least less than 0.05, Student’s *t* test) when compared with the corresponding time points for DMSO/saline injection. Mean ± SEM. In **b**, mean ± SEM, *P < 0.05, **P < 0.01, ***P < 0.001 vs. 0.5% CMC group at the same time point (Student’s *t* test). **c** Decreased blood glucose levels in *Lepr*^*db/db*^ mice after chronic p.o. administration of NPC43. Male 65-day-old *Lepr*^*db/db*^ mice were fasted for 2 h and then fed daily (by oral gavage) with 0.5% CMC or NPC43 for 5, 7 and 9 consecutive days. Blood glucose levels in 2 h-fasted *Lepr*^*db/db*^ mice before (day 0) and after p.o. treatment (day 5, 7 and 9) were determined using a glucometer. Mean ± SEM. *P < 0.05, **P < 0.01, ***P < 0.001 vs. 0.5% CMC group at the same time point (Student’s *t* test). **d**, **e** Decreased blood (**d**) glucose and (**e**) HbA1c levels in *Lepr*^*db/db*^ mice after chronic i.p. treatment with NPC43. Male *Lepr*^*db/db*^ mice at 38 days of age were i.p. injected daily with 0.2% (v/v) DMSO/physiological saline (saline control) or NPC43 (0.136 mg/kg BW) for 43, 52 or 90 days. Blood or sera from overnight-fasted saline- or NPC43-treated mice were subjected to (**d**) blood glucose and (**e**) HbA1c analysis. Data shown in **d** and **e** were collected from two independent animal experiments. Mean ± SEM. *P < 0.05, **P < 0.01 vs. saline control group (Student’s *t* test). **f** Improved glucose tolerance in *Lepr*^*db/db*^ mice after chronic i.p. treatment with NPC43. Male *Lepr*^*db/db*^ mice at 41-days of age were i.p. injected daily with 0.2% (v/v) DMSO/physiological saline or NPC43 (0.136 mg/kg BW) for 43 days, fasted overnight and then subjected to intraperitoneal glucose tolerance tests. Blood glucose levels (left panel) at indicated time point before or after i.p glucose injection in these mice were determined and the areas under the curve (right panel) were calculated. Numbers in parentheses in the left panel refer to the ratio of animals with blood glucose levels ≥ 600 mg/dl. Mean ± SEM. *P < 0.05, **P < 0.01 vs. saline control group at the same time point (Student’s *t* test). **g** Decreased serum insulin levels in *Lepr*^*db/db*^ mice after chronic i.p. treatment with NPC43. Male *Lepr*^*db/db*^ mice at 38-days of age were i.p. injected daily with 0.2% (v/v) DMSO/physiological saline or NPC43 (0.136 mg/kg BW) for 52 days. Sera from overnight-fasted saline- and NPC43-treated *Lepr*^*db/db*^ mice as well as age-matched wild-type C57 mice (without treatments, referred to normal group) were subjected to the insulin analysis. Numbers on the top of each column are the mean values of insulin levels. Mean ± SEM. Different letters represents a statistical significance between those two groups (Student’s *t* test)

The robust inhibition of glucose production from HepG2 cells by NPC43 (Fig. 1b) prompted us to investigate its anti-hyperglycemic potential in T2D *Lepr*^*db/db*^ mice [27]. First, the effective anti-hyperglycemic dose range and duration of action of NPC43, following a single i.p. injection into overnight-fasted adult male *Lepr*^*db/db*^ mice, were assessed. As shown in Fig. 2a, acute treatment with NPC43 over a 1000-fold dose range (0.0054–5.4 mg/kg BW) resulted in a marked decrease in blood glucose levels (when compared to a DMSO/saline control group) at each time-period beginning at 1-h post-NPC43-injection. NPC43 efficacy was maximal at 2–5 h post-i.p. administration, and the effect persisted for ≥ 3 h beyond this.

The anti-hyperglycemic effect of acute oral administration of NPC43 was also investigated in adult *Lepr*^*db/db*^ mice. As shown in Fig. 2b, oral administration of NPC43 (1.08 mg/kg BW) in *Lepr*^*db/db*^ mice elicited a significant decrease in blood glucose levels (when compared to a control vehicle group orally gavaged with 0.5% CMC) at each tested time-period beginning at 1 h post NPC43-treatment. The effect of NPC43 in lowering blood glucose levels in *Lepr*^*db/db*^ mice was maximal at 3–5 h post-administration, and lasted for at least 5 h. This indicates that NPC43 can be administered either orally or via injection to rapidly and effectively lower blood glucose levels in a model of severe insulin-resistance and T2D.

### Chronic i.p. and p.o. treatment with NPC43 reduced blood glucose levels in *Lepr*^*db/db*^ mice

Daily oral administration of two doses of NPC43 to *Lepr*^*db/db*^ mice for 9 days with blood glucose measurements at 5, 7 and 9 days resulted in significantly reduced glycemia when compared to the 0.5% CMC (w/v) control animals (Fig. 2c). Chronic i.p. treatment of this animal model with 0.2% (v/v) DMSO/saline (saline control group) or NPC43 (0.136 mg/kg BW daily for 43 or 90 days) was also investigated. We observed no animal mortality or abnormalities in mouse gross morphology and animal behavior in *Lepr*^*db/db*^ mice for the duration of this chronic NPC43 treatment. However, treatment with NPC43 for 43 days resulted in a 45% reduction in fasting blood glucose levels, relative to saline-treated controls (Fig. 2d). Treatment for 90 days brought about a greater than 50% reduction in fasting blood glucose and restored these animals almost to normoglycemia (~ 100 mg/dl) as reported for the normal C57BL/6J mouse strain [12]. Consistent with the reduced fasting blood glucose concentrations in response to NPC43, levels of the more retrospective glycemic indicator, HbA1c or glycated HbA1c, were also significantly reduced (20–30%) in response to the compound, relative to saline-treated animals (Fig. 2e).

### Attenuated glucose intolerance and hyperinsulinemia in *Lepr*^*db/db*^ mice after chronic i.p. treatment with NPC43

The glucose tolerance test detects dysfunctional glucose clearance after a high and rapid rise of blood sugar (e.g., after a meal). Like T2D sufferers, *Lepr*^*db/db*^ mice display impaired glucose tolerance and hyperinsulinemia [27]. Accordingly, the effect of chronic treatment of male *Lepr*^*db/db*^ mice with NPC43 on glucose tolerance was investigated. As shown in Fig. 2f, blood glucose levels in NPC43-treated *Lepr*^*db/db*^ mice, especially at 1 and 2 h after glucose challenge, were lower than saline-treated mice, and the blood glucose levels at 2 h post-glucose injection in NPC43-treated mice were almost restored to pre-challenge glucose concentration. Quantitation of the area under the curve (AUC) in these glucose tolerance tests showed that there was a significant decrease in AUC in NPC43-treated mice when compared to saline-treated mice (Fig. 2f, right panel).

Next, the effect of NPC43 on hyperinsulinemia was investigated. As shown in Fig. 2g, *Lepr*^*db/db*^ mice displayed hyperinsulinemia with serum insulin levels of 2586 μIU/ml. However, chronic i.p. treatment with NPC43 in *Lepr*^*db/db*^ mice resulted in an 80% decrease in serum insulin levels to 525 μIU/ml (Fig. 2g).

### Inhibition of *G6pc* expression in cultured liver cells and liver tissues of *Lepr*^*db/db*^ mice by NPC43

**Fig. 3:**
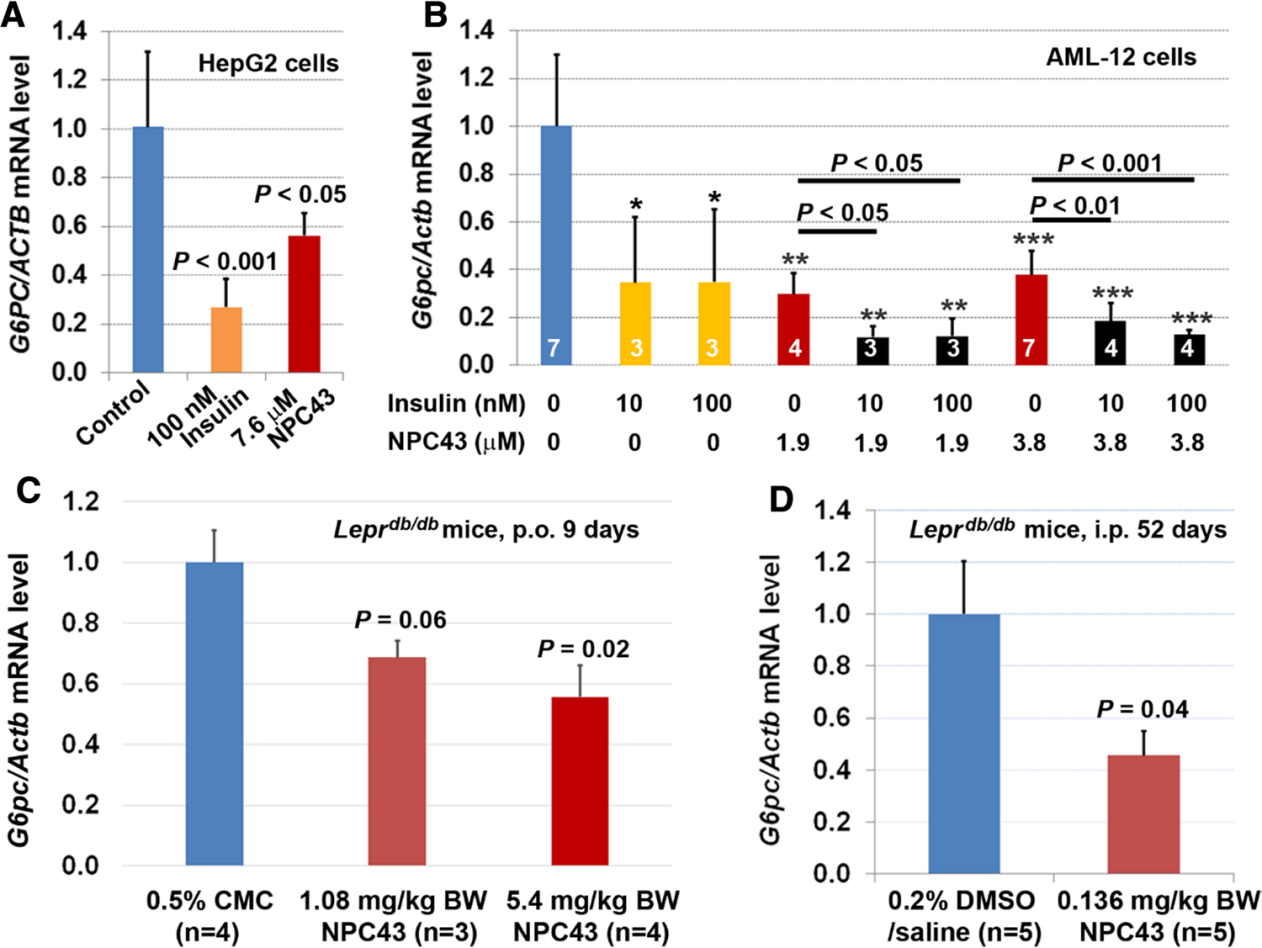
Inhibition of *G6PC/G6pc* expression by NPC43 in both human HepG2 and mouse AML-12 liver cells as well as in the liver of *Lepr*^*db/db*^ mice. **a** Insulin-mimetic activity of NPC43 in the inhibition of *G6PC* expression in human HepG2 cells. HepG2 cells were treated without (control) or with indicated concentrations of insulin or NPC43 in serum-free media for 40 h and then subjected to QRT-PCR analysis. Mean ± SD of four replicates per group and *P* value (vs. control) was determined by performing Student’s *t* test. **b** Insulin-mimetic activity of NPC43 in the inhibition of *G6pc* expression, and the cooperative action of both NPC43 and insulin in the inhibition of *G6pc* expression in mouse AML-12 cells. AML-12 cells were pretreated without or with NPC43 (1.9 or 3.8 μM) in FBS-containing but ITS/Dex-free media for 24 h. Then these AML-12 cells were treated without or with insulin (10 or 100 nM), NPC43 (1.9 or 3.8 μM) or both in serum/ITS/Dex-free media for 6 h, and subjected to QRT-PCR analysis. Data were obtained from two combined experiments and presented as mean ± SD of the indicated number (shown inside the column in the bar graph) of samples or replicates per group. *P < 0.05, **P < 0.01, ***P < 0.001 vs. non-insulin/NPC43 group (Student’s *t* test). Statistical results (Student’s *t* test) between NPC43-treated and both NPC43 and insulin-treated groups were also shown in the bar graph. **c** Reduced *G6pc* mRNA in the livers of *Lepr*^*db/db*^ mice after chronic oral administration of NPC43. *Lepr*^*db/db*^ mice at postnatal day 65 were fed (by oral gavage) with 0.5% CMC or NPC43 (dissolved in 0.5% CMC, 1.08 and 5.4 mg/kg BW) daily for 9 days. Liver tissues from 0.5% CMC- or NPC43-treated *Lepr*^*db/db*^ mice were collected and subjected to QRT-PCR analysis. Mean ± SEM. *P* values (vs. the 0.5% CMC group, Student’s *t* test). **d** Reduced *G6pc* mRNA in the livers of *Lepr*^*db/db*^ mice after chronic i.p. treatment with NPC43. *Lepr*^*db/db*^ mice at postnatal day 38 were i.p. injected with 0.2% DMSO/saline (v/v) or NPC43 (0.136 mg/kg BW) daily for 52 days. Liver tissues from these DMSO/saline- or NPC43-treated *Lepr*^*db/db*^ mice were collected and subjected to QRT-PCR analysis. Mean ± SEM. *P* value vs. the DMSO/saline group (Student’s *t* test)

Suppression of glucose production from hepatic cells and restoration of glycemic control in T2D mice suggested that glucose-6-phosphatase, which catalyzes the terminal step in gluconeogenesis and glycogenolysis and whose expression is inhibited by post-prandial insulin in normal liver cells [1, 28], may also be downregulated by the action of NPC43. This was shown to be the case in HepG2 cells cultured in serum-free media where NPC43, like insulin, elicited a significant decrease in *G6PC* mRNA expression (Fig. 3a). The same effect was noted in the mouse liver cell line, AML-12, where a significant downregulation of *G6pc* expression occurred in response to either insulin (10 or 100 nM) or NPC43 (1.9 or 3.8 μM) treatment (Fig. 3b). Of interest was the observation that cotreatment with insulin and NPC43 led to more pronounced downregulation of *G6pc* in AML-12 cells (Fig. 3b), raising the possibility that NPC43 can act as a potentiator of insulin action in suppressing glucose production from liver cells. However, the most important point is that the above results showed that, like insulin, NPC43 can inhibit *G6PC* or *G6pc* expression in cultured human and mouse liver cells.

These observations were confirmed in vivo, in *Lepr*^*db/db*^ mice, following chronic p.o. and i.p. treatment with NPC43. As shown in Fig. 3c, daily p.o. administration of NPC43 in *Lepr*^*db/db*^ mice at doses of 1.08 and 5.4 mg/kg/BW for 9 days caused a ~ 30% and ~ 45% reduction of *G6pc* mRNA levels in the liver, respectively. Similarly, chronic i.p. treatment with NPC43 (0.136 mg/kg/BW, daily for 52 days) resulted in a ~ 56% reduction of *G6pc* mRNA levels in the livers of *Lepr*^*db/db*^ mice (*vs* the saline-treated control, Fig. 3d).

### NPC43 activated the major components of the insulin signaling cascade in the liver

Forkhead box 1 (FOXO1) is the direct transcriptional regulator of *G6pc* expression in mammalian liver, and the activity of FOXO1 is tightly regulated by insulin/INSR/phosphoinositide-dependent kinase-1(PDK1)/protein kinase B (AKT) signaling [29, 30]. The robust decrease of hepatic *G6pc* expression in *Lepr*^*db/db*^ mice, human and mouse liver cells (Fig. 3) could be due to the ability of NPC43 to circumvent the requirement of insulin/INSR interactions, yet still activate INSR/PDK1/AKT signaling with subsequent inactivation of FOXO1 via increased FOXO1 phosphorylation. Thus, the expression of these signaling molecules was investigated in NPC43-treated HepG2 cells and *Lepr*^*db/db*^ mice, respectively.

**Fig. 4:**
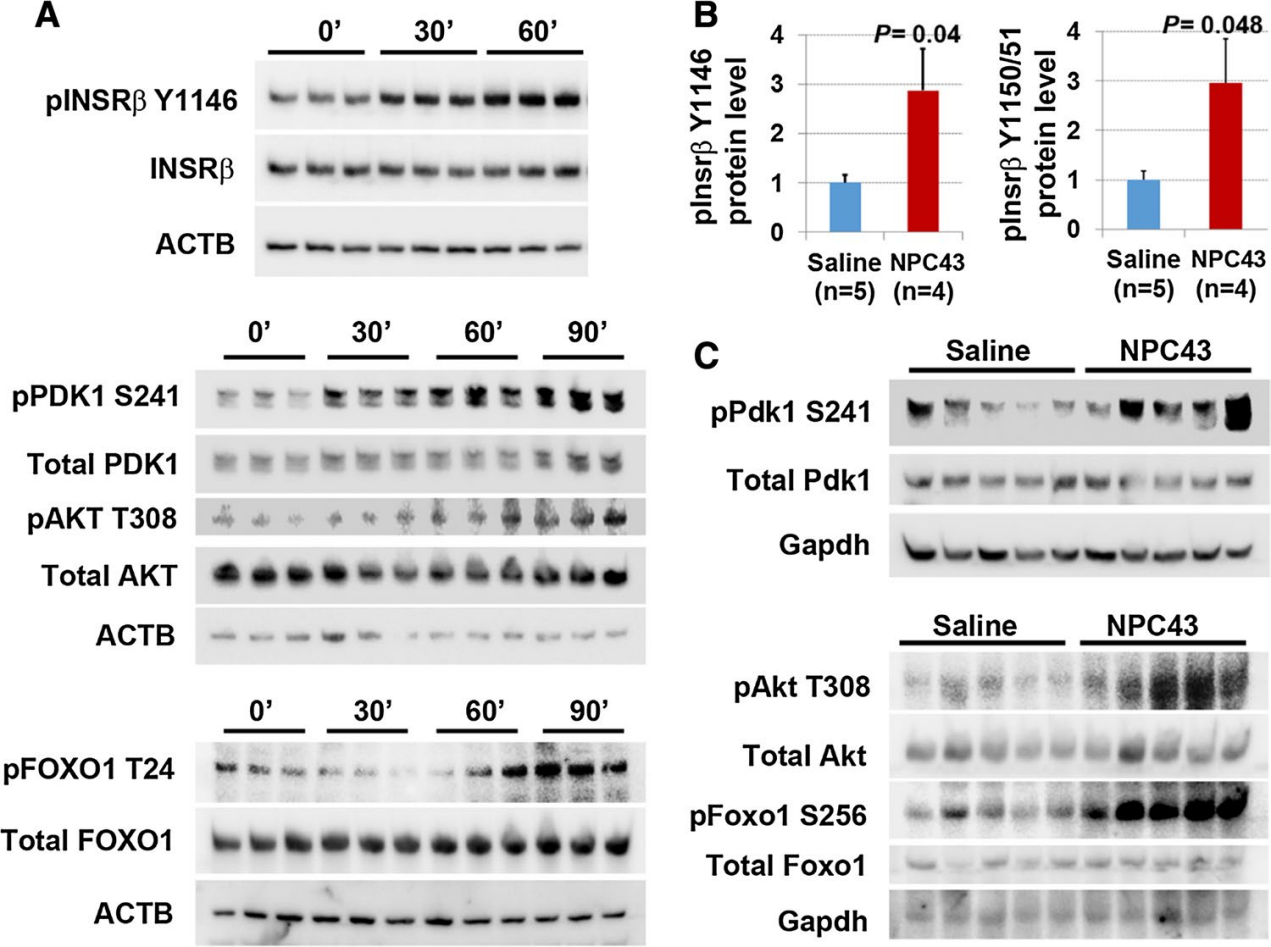
Insulin-independent activation of INSR signaling by NPC43 in both HepG2 cells and liver tissues of *Lepr*^*db/db*^ mice. **a** Enhanced phosphorylation of INSR, PDK1, AKT and FOXO1 in HepG2 cells after NPC43 treatments. HepG2 cells were serum-starved overnight, incubated without (0 min) or with NPC43 (7.6 μM) for 30 min, 60 min or 90 min (triplicates/group) and then subjected to Western blot analysis (using 5 μg protein per sample). Quantitative data for protein expression of these molecules (shown in Western blots) are shown in Online Resource 5. **b**, **c** Enhanced phosphorylation of (**b**) Insrβ at Y1146 and Y1150/1151 and (**c**) Pdk1/Akt/Foxo1 in the livers of *Lepr*^*db/db*^ mice after chronic i.p. treatment with NPC43. Five *Lepr*^*db/db*^ mice at postnatal day 38 were i.p. injected with 0.2% (v/v) DMSO/saline (saline control) or NPC43 (0.136 mg/kg BW) daily for 52 days. Liver tissues from these saline- and NPC43-treated *Lepr*^*db/db*^ mice were collected and then subjected to (**b**) ELISA analysis of phosphorylated Insrβ at Y1146 (using 400 μg protein/mouse) or at Y1150/1151 (using 600 μg protein/mouse) and (**c**) Western blot analysis of other listed molecules (using 100 μg protein/mouse). In **b**, data are presented as mean ± SEM of indicated numbers of animals and *P* values (vs. the saline group) were determined using the Student’s *t* test program. Quantitative data for protein expression of Pdk1/Akt/Foxo1 signaling molecules in **c** Western blots are shown in Online Resource 6

As shown in Western blots in Fig. 4a, treatment with 7.6 μM NPC43 in HepG2 cells for both 30 and 60 min in serum-free media resulted in a marked increase of phosphorylated-INSRβ (pINSRβ) at Y1146 (the marker for activated INSR [4]), but not total INSRβ. Quantitative analysis of protein band densities in these Western blots demonstrated that there was a significant increase in pINSRβ Y1146 protein levels in HepG2 cells after being treated with NPC43 for 30 and 60 min (Online Resource 5, panel A). Similarly, a marked increase in the protein levels of phosphorylated-PDK1 (pPDK1) at S241 was observed in HepG2 cells after NPC43 treatment for 30, 60 and 90 min (Fig. 4a and Online Resource 5, panel B). Protein levels of phosphorylated-AKT (pAKT) at T308 were also significantly increased in HepG2 cells after treatment with NPC43 for 60 and 90 min (Fig. 4a and Online Resource 5, panel C). In addition, increased FOXO1 phosphorylation was also observed in HepG2 cells after treatment with NPC43 for 90 min (Fig. 4a and Online Resource 5, panel D) and 24 h (data not shown). In contrast, there was no significant change in total PDK1, AKT or FOXO1 protein levels in HepG2 cells after treatment with NPC43 for ≤ 90 min (Fig. 4a and Online Resource 5, panel B–D). Thus NPC43 can indeed activate INSR/PDK1/AKT signaling, thereby eliciting FOXO1 phosphorylation in HepG2 cells.

These results were again confirmed in vivo where a significant increase (2.9-fold) in phosphorylation of Insrβ at Y1146, 1150 and 1151 was observed in the liver of *Lepr*^*db/db*^ mice after chronic i.p. treatment with NPC43 (Fig. 4b). Similarly, chronic i.p. treatment with NPC43 caused significant increases in the ratios of pPdk1, pAkt and phosphorylated-Foxo1 (pFoxo1) to total Pdk1, Akt and Foxo1 protein levels, respectively, in the livers of *Lepr*^*db/db*^ mice (Fig. 4c and Online Resource 6). NPC43 is thus capable of activating the main components of the insulin signal transduction pathway in insulin-resistant liver.

### NPC43 induces phosphorylation of AS160 (also known as TBC1D) and stimulates hepatic glucose import

Glucose transporter type-4 (GLUT4) translocation to the plasma membrane and a rapid increase in glucose transport in response to insulin follows phosphorylation of the GLUT4 transporter inhibitor, AS160, by AKT [31, 32]. The observed alternative activation of INSR/AKT signaling by NPC43 prompted us to investigate if this compound could elicit AS160 phosphorylation in normal and diabetic cell types and bring about an increase in glucose uptake.

**Fig. 5:**
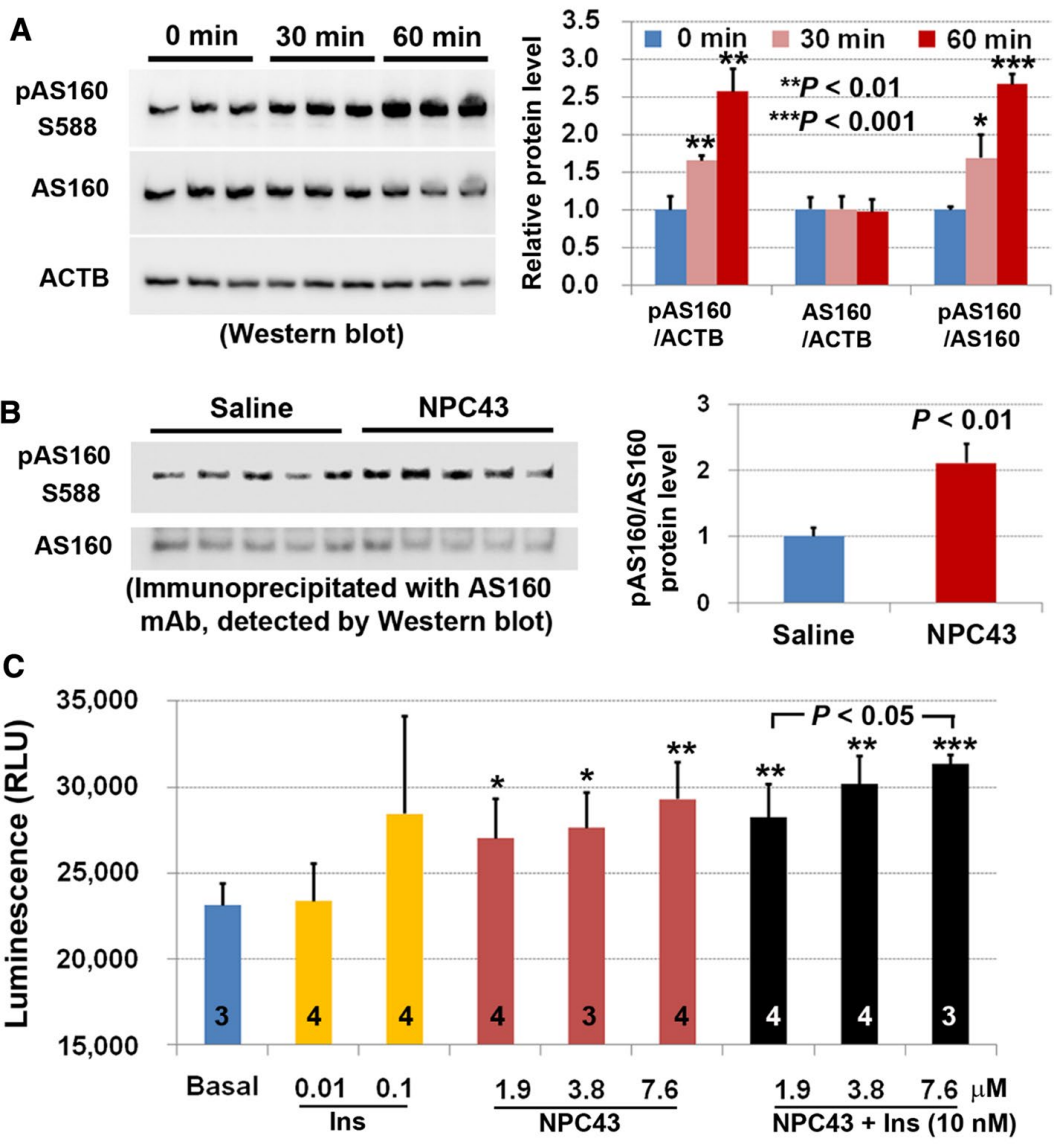
Enhanced AS160 phosphorylation in both human HepG2 cells and livers of *Lepr*^*db/db*^ mice and stimulation of glucose uptake in mouse AML-12 cells by NPC43. **a** Western blot analysis showing the increased AS160 phosphorylation in HepG2 cells after treatment with NPC43 (7.6 μM) in serum-free media for 30 and 60 min. Five micrograms of HepG2 cell protein extracts (triplicates/group) were used in Western blot analysis, and quantitative expression levels of these proteins in Western blots (left panel) were determined using NIH Image J software and are shown in the right panel. Mean ± SD. *P < 0.05, **P < 0.01, ***P < 0.001 vs. the 0 min (before NPC43 treatment) control group (Student’s *t* test). **b** Enhanced AS160 phosphorylation in the livers of *Lepr*^*db/db*^ mice after chronic treatment with NPC43 (0.136 mg/kg BW, i.p. daily for 52 days starting at postnatal day 38), as determined by immunoprecipitation followed by Western blot analysis. One milligram of liver protein extract from five individual mice after saline or NPC43 treatments was used in immunoprecipitation studies, and quantitative expression levels of these proteins in Western blots (left panel) were determined using NIH Image J software and are shown in the right panel. Mean ± SEM of five animals/group. *P* value vs. the saline group (Student’s *t* test). **c** Insulin-like activity of NPC43 in the stimulation of glucose uptake in AML-12 cells. AML-12 cells (1.5 × 10^5^/96-well) were cultured overnight, serum-starved overnight and then treated without (basal) or with insulin, NPC43 or both insulin (10 nM) and NPC43 in glucose- and phenol red-free DMEM media at 37 °C for 1.5 h followed by incubation with 1 mM 2-deoxyglucose (2DG) at room temperature for 30 min. Luminescence of up-taken glucose (i.e., 2DG) was determined using a luminometer. Data are presented as mean ± SD of the indicated number (shown inside the column in the bar graph) of replicates per group. *P < 0.05, **P < 0.01, ***P < 0.001 vs. the basal group (Student’s *t* test)

Treatment of HepG2 cells maintained in serum-free media with NPC43 (7.6 µM) for 30 and 60 min, respectively, caused significant increases in phosphorylated AS160 (pAS160) levels without affecting total AS160 protein concentration (Fig. 5a). Consistent with these results in HepG2 cells, chronic treatment with NPC43 (i.p. daily for 52 days) caused a 2.1-fold increase (*P* < 0.01) in protein levels of pAS160 in the liver of *Lepr*^*db/db*^ mice (Fig. 5b). Finally, the direct effect of NPC43 administration on glucose uptake was examined in mouse AML-12 liver cells cultured under serum-free conditions. As shown in Fig. 5c, treatment with NPC43 at concentrations of 1.9, 3.8 and 7.6 μM, respectively, resulted in significant increases, relative to basal levels, in glucose uptake by AML-12 cells. The extent of increased glucose uptake in AML-12 cells after treatment with 1.9-7.6 μM NPC43 was comparable to the 100 nM insulin treatment (~ 23% increase; Fig. 5c). Notably, although 10 nM insulin did not induce glucose uptake in AML-liver cells, co-administration of 10 nM insulin with NPC43 at 3.8 and 7.6 µM, respectively, enhanced glucose uptake to levels slightly higher than those observed with the compound NPC43 alone (Fig. 5c), thus possibly indicating cooperativity between insulin and NPC43 in stimulating glucose uptake in cultured liver cells.

### NPC43 activates Insr/Akt/AS160 signaling in differentiated mouse C2C12 cells and in skeletal muscle from *Lepr*^*db/db*^ mice

**Fig. 6:**
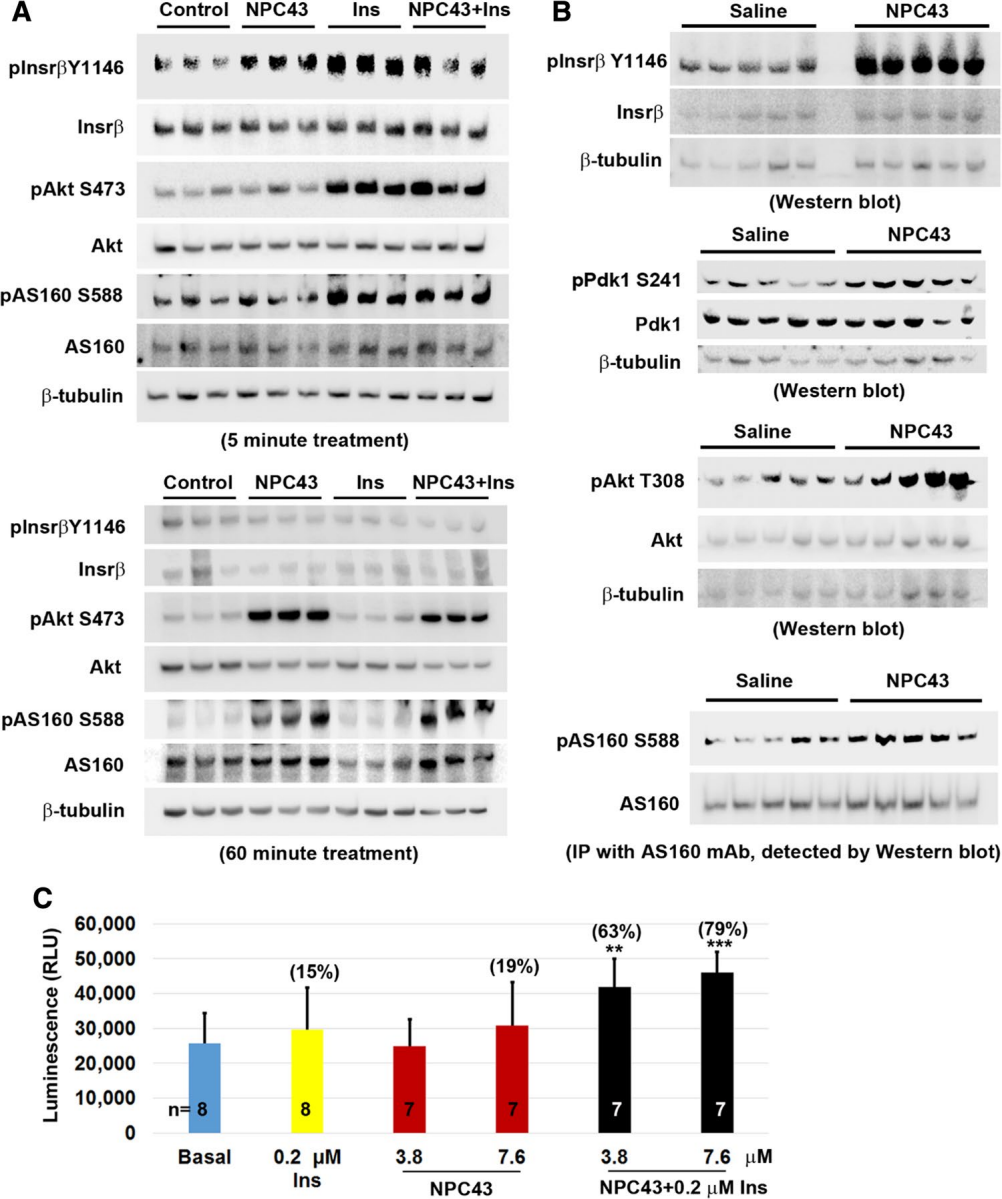
Activation of Insr/Akt/AS160 signaling in both differentiated mouse C2C12 cells and skeletal muscle of *Lepr*^*db/db*^ mice by NPC43 and cooperative action of NPC43 and insulin in the stimulation of glucose uptake in differentiated C2C12 cells. **a** Activation of Insr and stimulation of Akt and AS160 phosphorylation in differentiated mouse C2C12 (skeletal muscle) cells by NPC43. Completely differentiated C2C12 cells were serum-starved overnight, treated without (Control) or with NPC43 (7.6 μM), insulin (0.2 μM) or both in serum-free DMEM media at 37 °C for 5 (top panel) or 60 (bottom panel) minutes and then subjected to Western blot analysis (using 8 μg protein, triplicates/group). Quantitative data of the expression levels of these proteins detected in Western blots are shown in Online Resource 7. **b** Activation of Insr and stimulation of Akt and AS160 phosphorylation in the skeletal muscle of *Lepr*^*db/db*^ mice by NPC43. Five *Lepr*^*db/db*^ mice at postnatal day 38 were i.p. injected with 0.2% (v/v) DMSO/saline or NPC43 (0.136 mg/kg BW) daily for 52 days. Gastrocnemius from these mice was collected and subjected to Western blot analysis (using 100 μg protein/mouse) of pInsrβ Y1146 (activated Insr), pPdk1 S241 and pAkt T308 or immunoprecipitation (IP, using 400 μg protein/mouse) with a specific AS160 monoclonal antibody followed by Western blot analysis of pAS160 S588. Quantitative data for protein expression of the above Insr signaling molecules are shown in Online Resource 9. **c** Effects of insulin and NPC43 on glucose uptake in differentiated mouse C2C12 cells. C2C12 cells (5000 cells/96-well) were cultured in 10% FBS/DMEM media for 5 days and then differentiated using 0.5% horse serum/DMEM media for 7 days. These differentiated C2C12 cells were pretreated without or with NPC43 (3.8 or 7.6 μM) in serum- and glucose-free DMEM media overnight and then treated without (basal) or with insulin (0.2 μM), NPC43 (3.8 and 7.6 μM) or both insulin and NPC43 in serum- and glucose-free DMEM media at 37 °C for 1.5 h followed by incubation with 1 mM 2DG at room temperature for 30 min. Luminescence of up-taken glucose (i.e., 2DG) was determined using a luminometer. Data are presented as mean ± SD of indicated number of replicates per group. **P < 0.01, ***P < 0.001, vs. the basal group (Student’s *t* test). Percentage in parentheses in the bar graph refers to the mean percentage of increased glucose uptake in that group when compared to the basal group

Skeletal muscle is a critical insulin target organ which is responsible for 75% of whole body insulin-stimulated glucose uptake and the maintenance of normoglycemia [5, 33, 34]. The process is primarily mediated through INSR/AKT/AS160 signaling in response to insulin [31]. The observed activities of NPC43 against hyperglycemia and glucose intolerance in *Lepr*^*db/db*^ mice (Fig. 2) may also be attributed to NPC43-mediated activation of INSR/AKT/AS160 signaling in skeletal muscle to promote glucose uptake. Thus, the effect of NPC43 on the expression of these insulin signaling molecules was examined in differentiated mouse C2C12 (skeletal muscle) cells. As expected, treatment with insulin (0.2 μM) for 5 min, but not 60 min, resulted in a significant increase in protein levels of pInsrβ at Y1146, pAkt and pAS160 in C2C12 cells (Fig. 6a and Online Resource 7), indicating that these differentiated muscle cells are indeed responsive to insulin which transiently activates Insr/Akt/AS160 signaling. Importantly, treatment with NPC43 (7.6 μM) in serum-free media for 5 min also caused a significant increase in protein levels of pInsrβ at Y1146 in C2C12 cells (Fig. 6a and Online Resource 7, panel A). In addition, a robust increase in the levels of pAkt and pAS160 was observed in these cells after treatment with NPC43 for 60 min (Fig. 6a and Online Resource 7, panel B). Co-treatment with insulin and NPC43 for 5 min, but not 60 min, also tended to increase pInsrβ protein levels in these differentiated C2C12 cells (Fig. 6a and Online Resource 7). However, a significant increase in protein levels of pAkt and pAS160 was observed in these cells after co-treatment with NPC43 (7.6 μM) and insulin (0.2 μM) at both 5 and 60 min (Fig. 6a and Online Resource 7). The levels of increased Akt and AS160 phosphorylation in the co-treatment group at 5 and 60 min were comparable to insulin alone (at 5 min) and NPC43 alone (at 60 min), respectively (Fig. 6a and Online Resource 7). In contrast, treatment of differentiated C2C12 cells with 7.6 μM NPC43 for 6 h did not cause a significant increase in AS160 phosphorylation (Online Resource 8), indicating that NPC43 acts transiently to stimulate AS160 phosphorylation in skeletal muscle cells. Together, these results demonstrated that NPC43 can mimic insulin to quickly and transiently activate Insr, and subsequently enhance, albeit not as quickly as insulin does, the phosphorylation of Akt and AS160 in these differentiated C2C12 cells.

Next, the protein expression of Insr and its downstream signaling molecules, Pdk1, Akt and AS160, were examined in the gastrocnemius of *Lepr*^*db/db*^ mice after chronic i.p. treatment with NPC43. As shown in Fig. 6b and Online Resource 9 (panel A), protein levels of pInsrβ at Y1146 were significantly increased (~ 2.4-fold increase vs saline-treated control) in the skeletal muscle of NPC43-treated *Lepr*^*db/db*^ mice. In addition, the protein levels of pPdk1, pAkt and pAS160 were significantly increased in the skeletal muscle of *Lepr*^*db/db*^ mice post-NPC43 treatment, when compared to saline-treated mice (Fig. 6b and Online Resource 9, panel B–D). Together, these results demonstrate that NPC43 can activate Insr and its downstream signaling molecules, Pdk1, Akt and AS160 in the skeletal muscle of insulin-resistant, diabetic *Lepr*^*db/db*^ mice.

Finally, the ability of NPC43 to mimic and/or potentiate insulin action to enhance glucose uptake was investigated in differentiated C2C12 cells. As shown in Fig. 6c, treatment with NPC43 at a dose of 7.6 μM, but not 3.8 μM, tended to stimulate, albeit not significantly, glucose uptake (by ~ 19%) with a potency nearly comparable to 0.2 μM insulin (~ 15% increase) in these differentiated C2C12 cells. However, co-treatment with both NPC43 (3.8 μM) and insulin (0.2 μM) resulted in a significant 63% increase in glucose uptake by differentiated C2C12 cells (Fig. 6c). A more pronounced increase (79%) in glucose uptake was observed in these differentiated C2C12 cells after co-treatment with 7.6 μM NPC43 and 0.2 μM insulin (Fig. 6c).

### NPC43 treatments neither affected the activities of intracellular PTP1B and several major tyrosine kinases in vitro, nor activated IGF1R in liver and skeletal muscle of *Lepr*^*db/db*^ mice or in a cell-free, in vitro phosphorylation system

Enhanced phosphorylation of INSR and its downstream signaling molecules in HepG2 cells, differentiated C2C12 cells and liver and skeletal muscle of *Lepr*^*db/db*^ mice after NPC43 treatment (Figs. 4, 5, 6) could potentially be due to the inhibition of intracellular PTP1B activity. To test this, in vitro PTP1B assays were performed using pINSRβ [amino acid (aa) residues 1142–1153] at Y1146 as a substrate. As shown in Online Resource 10 (panel A), PTP1B activity was not significantly affected by NPC43 at all tested doses up to 100 μM. These results suggested that NPC43 did not directly inhibit intracellular PTP1B to enhance the phosphorylation of INSR and its downstream signaling molecules.

An in vitro tyrosine kinase screen was also performed to investigate whether NPC43 can enhance the activity of several major intracellular receptor tyrosine kinases. As shown in Online Resource 10 (panel B), the kinase activities of all tested intracellular receptor tyrosine kinases including INSRβ (aa 1101–1382), IGF1Rβ, HER4, KDR, PDGFRα and PDGFRβ were not stimulated by 50 µM NPC43.

Collateral activation of IGF1R is a key concern in the use of insulin or insulin analogs for the treatment of diabetes [35–37]. Thus, the effects of NPC43 on the activation of endogenous Igf1r were investigated in *Lepr*^*db/db*^ mice. As shown in Online Resource 10 (panel C, D), chronic treatment with NPC43 (0.136 mg/kg BW, i.p. daily for 52 days) in *Lepr*^*db/db*^ mice did not significantly increase the protein levels of pIgf1rβ at Y1131 in both liver and skeletal muscles, as determined by ELISA analysis. These results were further confirmed by co-immunoprecipitation studies (Online Resource 10, panel E, F). In addition, we also investigated whether NPC43 can directly activate IGF1R in a cell-free, in vitro phosphorylation system. As shown in Online Resource 10 (panel G), incubation of purified IGF1R proteins (containing both alpha and beta subunits) with 0.5 μM insulin induced the phosphorylation of IGF1R at Y1131. In contrast, NPC43 treatment at doses up to 7.6 μM did not stimulate IGF1R phosphorylation at Y1131 (Online Resource 10, panel G). These results suggest that NPC43 does not activate endogenous Igf1r or purified IGF1R proteins.

### In vitro interaction between NPC43 and INSR/Insr protein, and direct activation of rat liver Insr by NPC43 in a cell-free, in vitro phosphorylation system

**Fig. 7:**
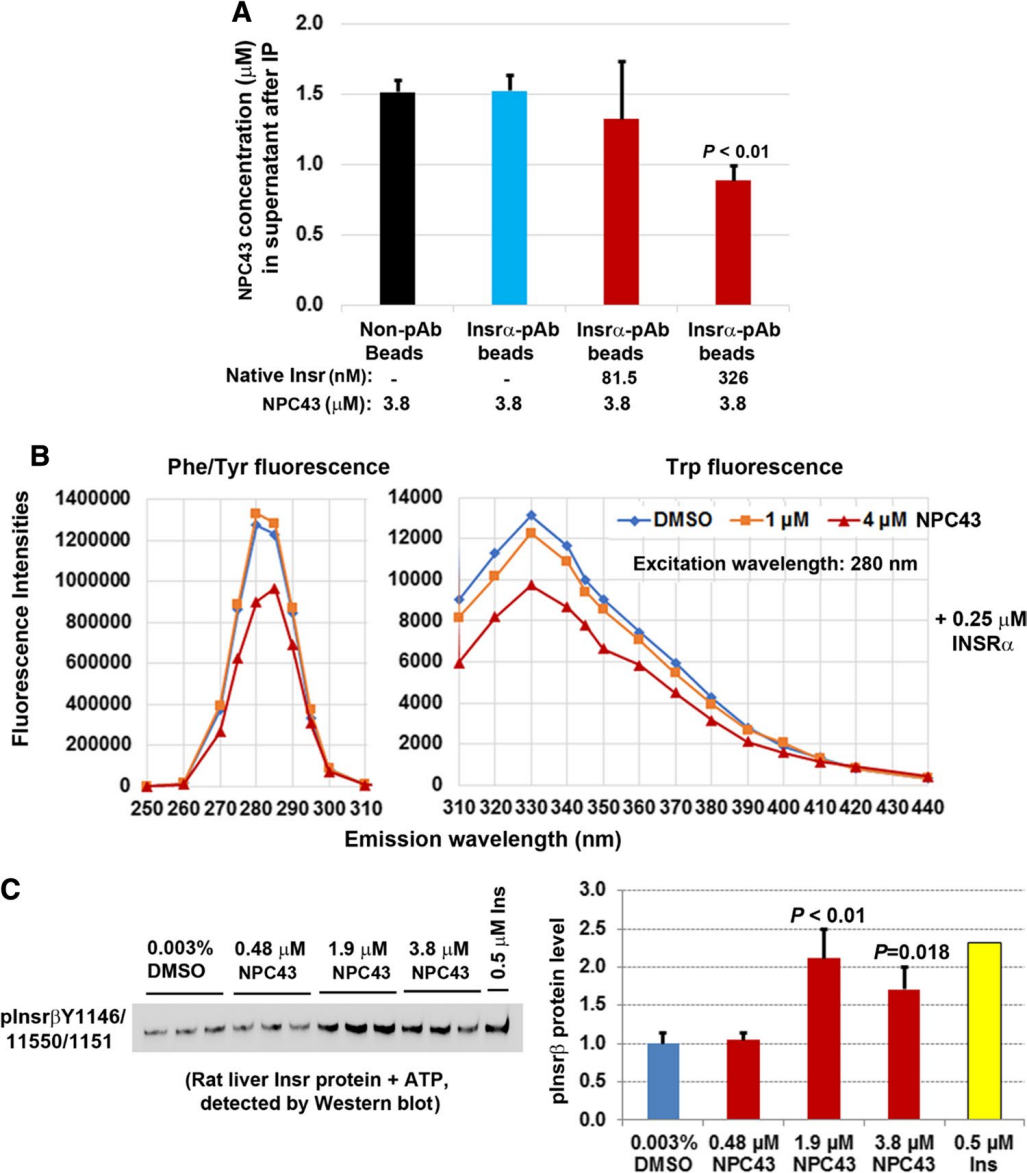
NPC43 interacted with the extracellular domain of INSR protein (INSRα) and directly activated rat liver Insr protein in a cell-free, in vitro phosphorylation system. **a** Cell-free in vitro binding of NPC43 to rat liver Insrα protein. NPC43 (3.8 μM) was incubated with or without native rat liver Insr proteins (containing Insrα and β) followed by incubation with non-antibody (Non-pAb)- or Insrα antibody (Insrα pAb)-conjugated magnetic beads [immunoprecipitation (IP)] to remove Insrα-bound NPC43 (by Insrα/Insrα-pAb-beads) or non-specific-bound NPC43 [by Non-pAb- or Insrα pAb-conjugated beads only (first and second column)]. The concentrations of remaining NPC43 in the supernatants after IP were determined by liquid chromatography coupled to time-of-flight mass spectrometry. Mean ± SD of triplicates per group. *P* value vs. the first and second columns in the bar graph (Student’s *t* test). **b** A representative fluorescent study showing a decrease in Phe/Tyr and Trp fluorescence in INSRα proteins after incubation with NPC43 (4 μM). NPC43 (1 or 4 μM) or its solvent DMSO (0.006%, v/v) in PBS buffer was incubated with 0.25 μM recombinant INSRα protein (aa 1–956) at room temperature for 10 min and then subjected to fluorescence analysis to obtain the fluorescence spectra of samples at the emission wavelengths from 250 to 440 nm with an excitation wavelength of 280 nm. Experiments were repeated three times. **c** Cell-free in vitro phosphorylation assays showing the direct activation of rat liver Insr proteins by NPC43. Equal amounts of purified rat liver Insr protein (final concentration: 0.13 μM) were incubated with 0.003% DMSO (triplicates), insulin (0.5 μM, singlet) or NPC43 (0.48, 1.9 or 3.8 μM, triplicates/group) in the presence of ATP, and the activated Insr (i.e. phosphorylated-Insrβ at Y1146, 1150 and 1151) was detected by Western blot analysis using both pInsrβY1146 and pInsrβY1150/1151 antibodies. Expression levels of activated Insr detected in the Western blot (left panel) were determined using NIH ImageJ software and are shown in the right panel. In the bar graph, data are presented as Mean ± SD of triplicates per group (except the insulin group) and *P* values were determined by performing Student’s *t* test (vs. the 0.003% DMSO group)

To investigate whether NPC43 can interact with Insrα in vitro, NPC43 (3.8 μM) was incubated without or with purified rat liver Insr protein (containing Insrα) and then immunoprecipitation using specific Insrα antibody-conjugated beads (equal amounts of beads/sample) was performed to remove Insrα-bound NPC43. Non-antibody (pAb)-conjugated magnetic beads were also incubated with NPC43 to determine the non-specific binding by the magnetic beads. The concentration of unbound NPC43 in the supernatant after immunoprecipitation was determined by liquid chromatography coupled with time-of-flight mass spectrometry. As shown in Fig. 7a, without incubation with rat liver Insr protein, NPC43 concentrations in the supernatants in the non-pAb bead group (after removal of beads) were the same as the Insrα-pAb-bead group, indicating that the Insrα pAb used did not bind to NPC43. Importantly, after incubation with increasing concentrations of native Insr proteins, NPC43 concentration in the supernatant was significantly decreased (Fig. 7a). These results indicate that NPC43 can bind to Insrα in vitro.

It has been reported that Tyr/Phe and Trp in proteins have intrinsic fluorescence at emission wavelengths (Em) of 280–304 nm and 330–340 nm [excited at (Ex) 280 nm], respectively, and that the fluorescence intensity of these amino acids is altered if the protein undergoes conformational changes [12, 26]. To further investigate whether NPC43 can directly interact with INSRα to elicit conformational changes in INSRα protein, the fluorescence spectra (at Em 250–440 nm/Ex 280 nm) of recombinant INSRα protein after incubation with NPC43 were recorded. As shown in Fig. 7b, a marked decrease in the intensities of Phe/Tyr fluorescence at Em 275–285 nm and Trp fluorescence at Em 310–370 nm was observed in recombinant INSRα protein after incubation with 4 μM NPC43. The magnitude of the decrease in Phe/Tyr fluorescence in recombinant INSRα protein after incubation with 4 μM NPC43 may actually be greater, considering that 4 μM NPC43 alone displayed intrinsic fluorescence at Em 270–290 nm/Em 280 nm (Online Resource 11, left panel) which overlapped Phe/Tyr fluorescence. At Em 310–440 nm/Ex 280 nm, NPC43 alone did not have intrinsic fluorescence (Online Resource 11, right panel), indicating that the observed decrease in Trp fluorescence in recombinant INSRα protein after incubation with 4 μM NPC43 could be wholly attributed to the conformational changes in INSRα protein. Together, these fluorescence studies further suggest that NPC43 can directly interact with INSRα protein, resulting in conformational changes of the insulin receptor.

Finally, in vitro phosphorylation assays were performed to investigate whether NPC43 can directly interact with and activate Insr in a cell-free system. As shown in Online Resource 12, addition of 0.5 μM insulin to rat liver Insr protein (containing both alpha and beta subunits of Insr) induced Insrβ phosphorylation at both Y1146 and Y1150/1151. Like insulin, incubation of rat liver Insr protein with NPC43 at doses of 3.8 and 7.6 µM also markedly induced Insrβ phosphorylation at Y1146 and 1150/1151 (Online Resource 12). To further confirm this observation and gain insight into the effective dose of NPC43, an equal amount of rat liver Insr protein was incubated with NPC43 at doses of 3.8 μM or lower in the in vitro phosphorylation system, and activated Insr in the samples was detected by both pInsrβY1146 and pInsrβY1150/51 antibodies. As shown in Fig. 7c, NPC43 treatment at doses of 1.9 and 3.8 µM, but not 0.48 μM, caused a significant increase in the level of pInsrβ at Y1146/1150/1151. Together, these data provide further evidence that NPC43, even at a low dose of 1.9 μM, can directly interact with and activate insulin receptor in the total absence of its natural ligand, insulin.

## Discussion

There is a clear need for an alternative to insulin and insulin analogs in the treatment of diabetes. The costs for insulin treatment are becoming unaffordable, even in developed countries, and the seemingly inexorable global increase in the number of diabetes sufferers who require insulin represents a genuine crisis for many health care systems [18–20]. An ideal replacement would be a stable, orally administered insulin receptor agonist with no side effects. We believe the findings presented here which describe a non-peptidyl, small molecule insulin mimetic represent a very significant step towards this goal.

Our initial screening system, based on the inhibition of glucose production in HepG2 cells, was used to assess a number of candidate compounds generated as a result of previous studies in our laboratory (2017, US patent number 9,642,874). One candidate in particular, NPC43; adenosine 5′-Se-methyl-5′-seleno-, 2′,3′-diacetate, inhibited glucose production much more potently than any other candidate including those which were structurally very similar (Fig. 1). In NPC43, the selenium atom is critical for the inhibition of glucose production in HepG2 cells, since the replacement of the selenium atom in NPC43 by a sulfur atom (Compound #68) resulted in only a slight decrease in glucose production (Fig. 1b, left panel). However, NPC43’s activity cannot be solely attributed to the selenium atom, as evidenced by the fact that Compound #E, another selenium-containing compound, did not inhibit glucose production and chemically similar selenium-bearing small molecules such as Compounds #C, #D, #50, #53, #69 and #70 exhibited widely variable inhibitory effects on glucose production (Fig. 1b, left panel). Structural comparison of Compounds #43, #C, #50, #53, #69, and #70 indicates that a diacetyl ester at 2′ and 3′ position of NPC43 is important for efficient inhibition of glucose production, since the replacement of this diacetyl ester by hydrogen molecules (Compound #C), cyclic carbonate (Compound #50), morpholino carboxylate (Compound #53), dipropanoyl ester (Compound #69), or dibutanoyl ester (Compound #70) resulted in much weaker inhibition of glucose production in HepG2 cells (Fig. 1b). Therefore, NPC43 is a novel insulin mimetic with a chemical structure (containing a selenium atom and a diacetyl ester at 2′ and 3′ position on the adenosine backbone) that is totally different from previously reported insulin mimetics [9–16].

In agreement with the inhibition of glucose production by NPC43 in HepG2 cells (Fig. 1), both acute and chronic treatment with NPC43, through i.p. and p.o. administration routes, respectively, resulted in a robust decrease in blood glucose levels in *Lepr*^*db/db*^ mice (Fig. 2a–e). The amelioration of glucose intolerance in *Lepr*^*db/db*^ mice after chronic treatment with NPC43 (Fig. 2f) further indicates the potential of NPC43 in the treatment of postprandial hyperglycemia. In addition, the markedly decreased blood insulin levels in NPC43-treated *Lepr*^*db/db*^ mice (Fig. 2g) may be a direct result of decreased blood glucose levels lowering the insulin production burden on β-cells in the pancreas. While further studies are needed to examine the detailed effect of NPC43 on insulin secretion from the pancreas, our results clearly demonstrated that NPC43 is an effective oral and injectable agent for the treatment or mitigation of hyperglycemia in insulin-resistant T2D subjects.

**Fig. 8:**
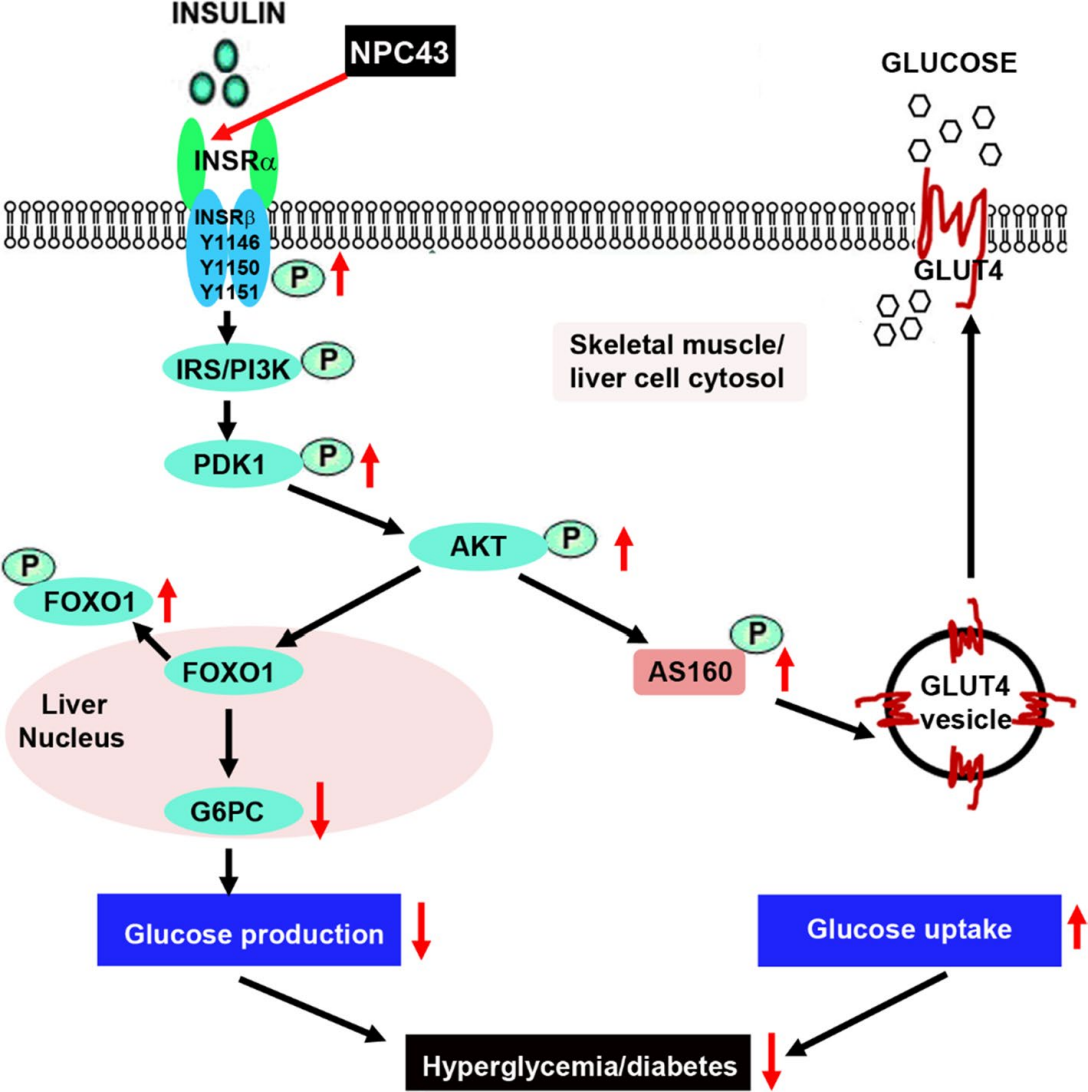
The mode of action of NPC43 against hyperglycemia and glucose intolerance in diabetes. NPC43 acts as a novel insulin-mimetic small molecule which interacts with extracellular INSRα to selectively activate INSR signaling in both liver and skeletal muscle cells. This leads the inhibition of glucose production and stimulation of glucose uptake. In addition, NPC43 also can cooperate with insulin to further inhibit *G6pc* expression in hepatocytes and enhance glucose uptake in skeletal muscle cells. More importantly, in insulin-resistant T2D subjects, NPC43 is able to restore INSR function in both liver and skeletal muscle, thereby attenuating hyperglycemia and glucose intolerance

Evidence that the anti-hyperglycemic activity of NPC43 (Fig. 2) is partly due to the attenuation of glucose production and the stimulation of glucose uptake in the liver resulting from the activation of INSR signaling by NPC43 in insulin-resistant *Lepr*^*db/db*^ mice (Fig. 8) is based on several observations. First, enhanced phosphorylation of Insr (represented by pInsrβ at Y1146) and its downstream signaling molecules, Pdk1, Akt, Foxo1 and AS160 as well as decreased expression of *G6pc* (a Foxo1 target gene which is critical for gluconeogenesis) were observed in the liver of *Lepr*^*db/db*^ mice after NPC43 treatment (Figs. 3c, d, 4b, c, 5b and Online Resource 6). Consistent with these findings in vivo, NPC43 was able to perform the role of insulin by directly activating INSR and inducing the phosphorylation of PDK1, AKT, FOXO1 and AS160 in HepG2 cells (Figs. 4a, 5a and Online Resource 5). In addition, the insulin-like action of NPC43 in the inhibition of basal *G6PC* or *G6pc* expression was clearly evident in cultured human and mouse liver cells (Fig. 3a, b). Furthermore, NPC43 mimicked the role of insulin by significantly inhibiting glucose production in HepG2 cells (Fig. 1b) and stimulating glucose uptake in mouse liver AML-12 cells (Fig. 5c). In all of our studies, therefore, NPC43 acted as a novel insulin mimetic in the liver to directly activate INSR signaling, thereby inhibiting glucose production and enhancing glucose uptake to mitigate hyperglycemia and glucose intolerance. Moreover, it is apparent that in addition to correcting insulin resistance, NPC43 can cooperate with insulin to further inhibit hepatic gluconeogenesis and stimulate liver glucose uptake, as indicated by the more conspicuous decrease in *G6pc* expression and the more evident increase in glucose uptake in mouse liver AML-12 cells after co-treatment with NPC43 and insulin rather than NPC43 or insulin alone (Figs. 3b, 5c).

Skeletal muscle functions as the largest site for glucose uptake [33, 38]. As such, changes to skeletal muscle health can severely impact whole-body glucose homeostasis. We present evidence that NPC43 also targets skeletal muscle in diabetic situations and functions in this tissue as an insulin mimetic, activating Insr and its downstream Pdk/Akt/AS160 signaling which leads to enhanced skeletal muscle glucose uptake, lower blood glucose levels and the promotion of normoglycemia (Fig. 8). Supporting data for this model include significant increases in the protein levels of activated Insr (represented by pInsrβ at Y1146), pAkt and pAS160 in the skeletal muscle of *Lepr*^*db/db*^ mice in response to chronic (52 days) i.p. treatment with NPC43 (Fig. 6b and Online Resource 9). Further corroboration of these in vivo data includes the observation that NPC43 can mimic insulin by quickly activating Insr and subsequently inducing the phosphorylation of Akt and AS160 in differentiated C2C12 cells (Fig. 6a and Online Resource 7).

Although NPC43 activates the same signaling pathway as insulin for the establishment of euglycemia, there are potentially important temporal differences in the phosphorylation of key insulin cascade components in skeletal muscle. Thus, while insulin-induced phosphorylation of Akt and AS160 has subsided by 60 min post-treatment in C2C12 cells, pAkt and pAS160 are still clearly evident in NPC43-treated and NPC43 + insulin-treated cells (Fig. 6a and Online Resource 7). Glucose uptake experiments in C2C12 cells treated with insulin alone or insulin in combination with NPC43 (Fig. 6c) illustrate an additional and potentially valuable attribute of NPC43, in the context of its slower activation of INSR downstream signaling molecules. Cells treated with insulin (0.2 µM) or NPC43 (7.6 µM) alone showed only a mild (15–19%) increase in glucose uptake relative to basal conditions (Fig. 6c). The lower concentration of NPC43 (3.8 µM) provoked no measurable glucose uptake (Fig. 6c). However, co-treatment with hormone plus the compound elicited a notably more potent response: a 63% increase (*P* < 0.01) over basal levels with 3.8 µM NPC43 and an 79% increase (*P* < 0.001) when 7.8 µM NPC43 was used (Fig. 6c). Such amplification and prolongation of INSR downstream signaling in skeletal muscle cells, when the hormone is used in combination with NPC43 (Fig. 6a, c), could significantly improve post-prandial glucose tolerance by allowing greater clearance of the sugar into muscle.

The mechanism by which NPC43 activates INSR and its downstream signaling molecules in liver and skeletal muscle, both in vivo and in vitro, is unlikely to be due to the inhibition of intracellular PTP1B, as previously reported for a chaetochromin-containing insulin-mimetic 4548-G05 [12]. This is because NPC43 at doses up to 100 μM failed to inhibit PTP1B activity in vitro (Online Resource 10, panel A). Also, unlike quinone-containing insulin-like compounds [9, 16], NPC43 did not directly activate intracellular INSRβ, as indicated by the in vitro tyrosine kinase analysis (Online Resource 10, panel B). We also performed in vitro phosphorylation analysis by incubating recombinant INSRβ (aa 999–1362) protein with NPC43 (3.8 and 7.6 μM) and did not observe any effect of NPC43 on INSRβ protein phosphorylation (unpublished data). Instead, NPC43 had a direct effect on the activation of INSR through interaction with the extracellular INSRα domains. Several lines of evidence lead to this conclusion, including: (a) incubation of increasing concentrations of rat liver Insr protein (containing Insrα) with NPC43 (3.8 μM), followed by removal of Insrα-bound NPC43, resulted in a significant decrease of measured NPC43 in the supernatant (Fig. 7a), indicating that NPC43 can physically bind to Insrα in vitro; (b) a marked decrease in the intensities of both Tyr/Phe and Trp fluorescence was observed in recombinant INSRα protein (aa 1–956) after incubation with 4 μM NPC43 (Fig. 7b), which further indicates that NPC43 can directly interact with INSRα, resulting in conformational changes in the INSRα protein structure; and (c) rat liver Insr protein, but not recombinant INSRβ protein (data not shown), was directly activated by NPC43 in a cell-free, in vitro phosphorylation assay system (Fig. 7c, Online Resource 12), further indicating that NPC43 can mimic insulin by directly activating INSR. This also demonstrates that the extracellular domain of INSR is required for NPC43 activation of the receptor.

The fluorescence studies (Fig. 7b) also suggest that aa residues such as Tyr, Phe and Trp in INSRα are involved in NPC43 binding, leading to a conformational change in the INSRα protein and activation of INSR. Although both NPC43 and insulin can directly interact with Insrα and activate Insr in the cell-free, in vitro phosphorylation system, the exact NPC43 binding sites in INSRα are most likely to be different from the reported insulin binding motifs [2]. In support of this, it was found that NPC43 robustly activated Insr in the liver and skeletal muscle of *Lepr*^*db/db*^ mice (Figs. 4b, 6b) displaying extreme hyperinsulinemia (Fig. 2g). In addition, the cooperative action between insulin and NPC43 in inhibiting *G6pc* expression in AML-12 cells (Fig. 3b) and stimulating glucose uptake in differentiated C2C12 cells (Fig. 6c) also suggests that NPC43 does not share the same binding sites in INSRα as insulin. Future studies such as insulin/NPC43-INSRα competition binding assays and INSRα protein mutation analysis are needed to precisely determine the NPC43 binding sites in INSRα. Regardless, our results demonstrate that the mechanism of NPC43 in the activation of INSR signaling is initiated through its direct interaction with INSRα, and that NPC43 is a novel INSRα agonist.

NPC43 is a fast-acting oral and injectable small molecule against hyperglycemia in T2D, as indicated by the rapid and significant decrease in blood glucose levels in *Lepr*^*db/db*^ mice at 1 h after i.p. and p.o. administration (Fig. 2a, b). This is further supported by the prompt activation of INSR (within 5–30 min) by NPC43 in cultured liver and skeletal muscle cells (Figs. 4a, 6a). Although the pharmacokinetics of NPC43 in *Lepr*^*db/db*^ mice remains to be characterized, the effectiveness of NPC43 in lowering blood glucose levels in these *Lepr*^*db/db*^ mice after chronic daily i.p. and p.o. treatment and the observed effective duration of 5–8 h after acute i.p. or p.o. treatment (Fig. 2a–e) indicates that NPC43 likely has a half-life in the range of hours, and that t.i.d. or even q.d. oral administration may be feasible in the use of NPC43 to treat hyperglycemia in humans. In addition, NPC43 appears to have a broad functional dose range against hyperglycemia, as indicated by the 1000-fold effective dose range (0.0054–5.4 mg/kg BW) observed in acute i.p. studies in *Lepr*^*db/db*^ mice (Fig. 2a).

Unlike previously identified quinone-containing insulin mimetics [15, 16], NPC43 did not enhance the kinase activity of several major intracellular receptor tyrosine kinases such as IGF1Rβ, HER4, KDR, PDGFRα and PDGFRβ in vitro (Online Resource 10, panel B), indicating that NPC43 is unlikely to trigger side-effects resulted from activation of these intracellular receptor kinases. Since collateral activation of IGF1R (implicated in increased cancer rates) is a key concern in the use of insulin or insulin analogs for the treatment of diabetes [35–37] and quinone-containing and polyphenolic insulin mimetics, such as DAQ-B1 and 6CI-TGQ, were previously reported to either activate or interact with IGF1R [14, 16], we performed a detailed analysis of activation status of endogenous Igf1r in liver and skeletal muscle of *Lepr*^*db/db*^ mice following daily i.p. NPC43 treatment for 52 days. Our results demonstrated that NPC43 did not activate endogenous Igf1r in liver and skeletal muscle of *Lepr*^*db/db*^ mice (Online Resource 10, panel C–F) in which endogenous Insr was markedly activated (Figs. 4b, 6b). In agreement with these in vivo observations, NPC43, unlike insulin, did not directly activate purified IGF1R protein (containing both alpha and beta subunits) in the in vitro phosphorylation assay system (Online Resource 10, panel G). These results suggest that NPC43, even after long-term use, does not activate IGF1R and thus is unlikely to elicit unwanted side-effects resulting from nonspecific IGF1R activation, such as those reported for some antidiabetic therapies, including insulin and its analog glargine [35, 37]. Furthermore, NPC43 at the tested doses did not display cytotoxicity to cultured human, rat and mouse liver cells, and chronic treatment with NPC43 did not cause an increase in serum ALT levels (an indicator of liver damage) in *Lepr*^*db/db*^ mice (Online Resource 4). Instead, there was a significant decrease in serum ALT levels in *Lepr*^*db/db*^ mice after chronic NPC43 treatment (Online Resource 4), indicating that NPC43 may have a protective effect against liver damage in diabetic subjects. In addition, chronic i.p treatment with 0.136 mg/kg BW NPC43 for up to 90 days did not cause any animal mortality or abnormalities in mouse gross morphology and animal behavior in *Lepr*^*db/db*^ mice. Also, preliminary toxicological/animal survival studies showed that oral administration of NPC43 at a dose of 15 mg/kg BW to Sprague–Dawley rats for 14 days or acute i.p. administration of NPC43 at the dose of 54 mg/kg BW into wild-type C57 and *Lepr*^*db/db*^ male mice did not result in any mortality in both animal species (unpublished data). These results further indicate that NPC43 likely has little cytotoxicity to the liver and other main organs to affect the general health. Moreover, as discussed earlier, the dramatic decrease in blood insulin levels of *Lepr*^*db/db*^ mice after chronic treatment with NPC43 (Fig. 2g) suggests that NPC43 could be of benefit in preserving β-cell function in the pancreas.

In summary, our results demonstrate that NPC43 is a novel, non-peptidyl insulin-mimetic small molecule with potent anti-diabetic activity. Unlike insulin, insulin analogs and the quinone-containing insulin mimetics [9, 15, 16], NPC43 can selectively activate INSR, but not IGF1R, in both liver and skeletal muscle, even in insulin-resistant subjects. Also, unlike the recently identified chaetochromin-derived insulin-like compound 4548-G05 [12], NPC43 does not inhibit intracellular PTP1B activity. Therefore, NPC43, through its interaction with INSRα and selective activation of INSR but not IGF1R, may represent a novel oral, safe and effective agent for the treatment of diabetes in humans.

## Electronic supplementary material

Below is the link to the electronic supplementary material.

Supplementary material 1 (PDF 80 kb)

Supplementary material 2 (PDF 108 kb)

Supplementary material 3 (PDF 41 kb)

Supplementary material 4 (PDF 158 kb)

Supplementary material 5 (PDF 166 kb)

Supplementary material 6 (PDF 131 kb)

Supplementary material 7 (PDF 242 kb)

Supplementary material 8 (PDF 137 kb)

Supplementary material 9 (PDF 162 kb)

Supplementary material 10 (PDF 282 kb)

Supplementary material 11 (PDF 197 kb)

Supplementary material 12 (PDF 162 kb)
