# Supplementary material for: Non-peptidyl small molecule, adenosine, 5′-Se-methyl-5′-seleno-, 2′,3′-diacetate, activates insulin receptor and attenuates hyperglycemia in type 2 diabetic Leprdb/db mice
Source: Cell Mol Life Sci. 2019 Aug 5;77(8):1623–43. doi: 10.1007/s00018-019-03249-4 (PMC7162833; doi:10.1007/s00018-019-03249-4)
Supplement: Supplementary file 1 — Supplementary material 1 (PDF 80 kb) [file 18_2019_3249_MOESM1_ESM.pdf]

Title: Non-peptidyl small molecule, adenosine, 5'-Se-methyl-5'-seleno-, 2',3'-diacetate, activates insulin receptor and attenuates hyperglycemia in type 2 diabetic *Lepr*$^{db/db}$ mice

Journal: Cellular and Molecular Life Sciences

Authors: Zi-Jian Lan, Zhenmin Lei, Alexandros Yiannikouris, Thirupathi Reddy Yerramreddy, Xian Li, Hayley Kincaid, Katie Eastridge, Hannah Gadberry, Chloe Power, Rijin Xiao, Lei Lei, Olivia Seale, Karl Dawson and Ronan Power

Correspondence to: zlan@alltech.com and rpower@alltech.com

Supplementary Material-1

Synthesis of NPC43 (Adenosine, 5'-*Se*-methyl-5'-seleno-, 2',3'-diacetate)

Scheme:

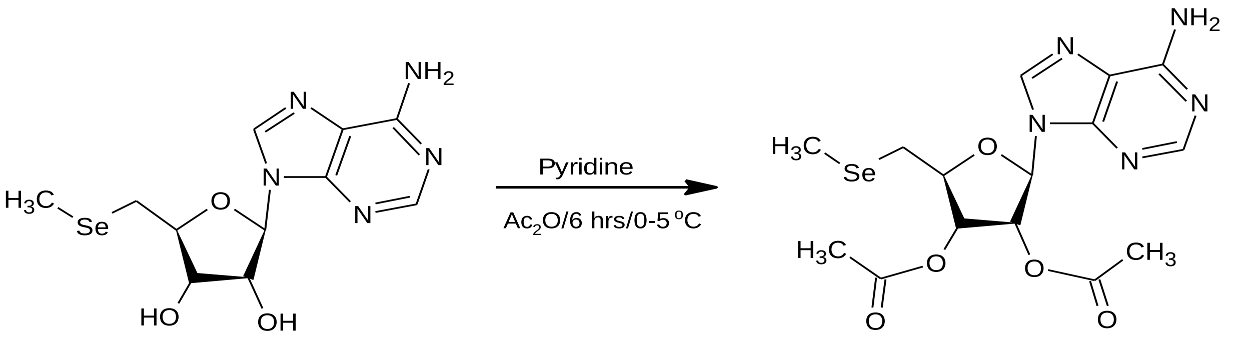

Synthetic procedure: 5'-*Se*-methyl-5'-seleno-Adenosine (1.0 gr, 0.0029 mole, 1.0 mole eq.) and anhydrous pyridine (10 ml) were placed in an oven dried, 50 ml three neck flask, equipped with a dropping funnel, inert gas inlet/outlet and a thermometer. The reaction set was placed in an ice/salt bath and agitation was initiated. When the temperature of the solution dropped to 0°C, acetic anhydride (10 ml, 0.105 mole, 36.47 mole eq.) was added drop-wise for 15 minutes and the temperature of the reaction mixture was maintained below 5°C during acetic anhydride addition. The reaction mixture was stirred for 6 hours at 0-5°C. The excess acetic anhydride was quenched by adding ice-cold water (100 ml), and then pH adjusted to 7 by adding 10% (wt/vol) $NaHCO_3$ aqueous solution. The aqueous mixture was extracted with ethyl acetate (2 x

100 ml). The combined ethyl acetate extracts were dried over anhydrous Na_2SO_4 (1gr) and filtered into a 250 ml round-bottomed flask. The filtrate was concentrated to dryness under reduced pressure at 35-40°C to yield a crude product as a pale yellow syrupy liquid. Pure product was then obtained as off-white crystals (1.12 gr, Yield: 90.3 %, Purity by HPLC and mass spectrometry: >99%) by passing the yellow liquid through a silica gel column with a mixture of ethyl acetate and hexanes (1:3 v/v).

Synthesis of Adenosine, 5'-*Se*-methyl-5'-seleno-, cyclic 2',3'-carbonate (Compound #50):

Scheme:

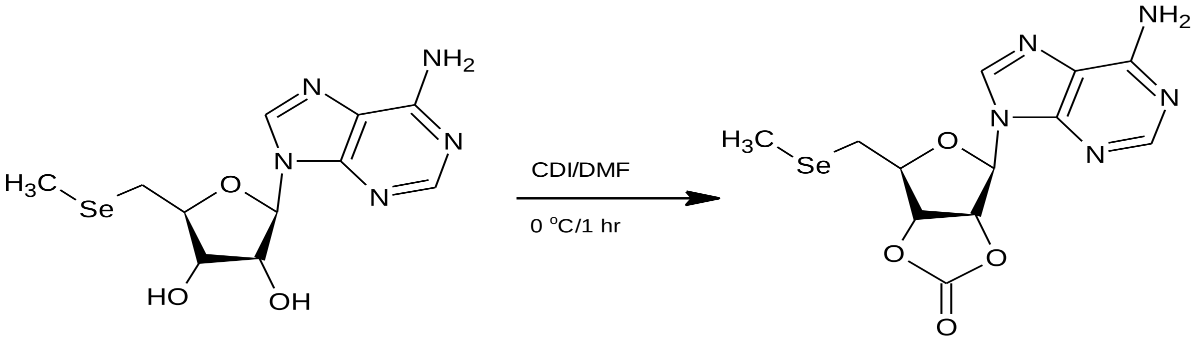

Synthetic procedure: 5'-*Se*-methyl-5'-seleno-Adenosine (1.0 gr, 0.0029 mole, 1.0 mole eq.) and anhydrous dimethylformamide (20 ml) were placed in an oven dried, 50 ml three neck flask, equipped with a dropping funnel, inert gas inlet/outlet and a thermometer. The reaction set was placed in an ice/salt bath and agitation was initiated. When the temperature of the solution dropped to 0°C, carbonyldiimidazole (CDI, 0.57 gr, 0.0035 mole, 1.21 mole eq.) was added at below 5°C. The reaction mixture was slowly warmed to the room temperature, and then stirred the reaction mixture for 4 hours at the same temperature under argon gas atmosphere. The solvent was removed under reduced pressure to yield a residue, which was then dissolved in a mixture of chloroform (5 ml) and ethanol (few drops) to get clear solution. The organic layer was washed with 1% aq. acetic acid solution (2 x 1 ml) and dried over anhydrous Na_2SO_4 (1 gr), filtered into a 250 ml round-bottomed flask. The filtrate was concentrated to dryness under reduced pressure at 25-30°C to yield a crude product as a pale yellow syrupy liquid. The crude product was dissolved in a mixture of ethanol/water mixture (1:1 v/v), and then concentrated to dryness under reduced pressure at 45-50°C to give a residue to which hexane (25 ml) was added

and stirred for 10 minutes. The mixture was concentrated to dryness under reduced pressure at 30-35°C to yield the desired product as a off-white crystals (1.02 gr, Yield: 95.3 %, Purity by HPLC and mass spectrometry: >99%).

Synthesis of a regio-isomeric mixture of Adenosine, 5'-*Se*-methyl-5'-seleno-, 2'-(4-morpholinocarbamate) and Adenosine, 5'-*Se*-methyl-5'-seleno-, 3'-(4-morpholinocarbamate) (Compound #53):

Scheme:

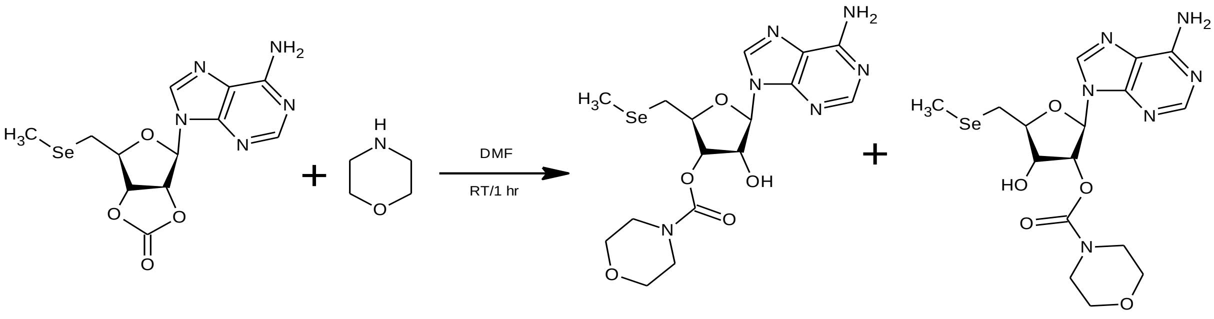

Synthetic procedure: Adenosine, 5'-*Se*-methyl-5'-seleno-, cyclic 2',3'-carbonate (1.0 gr, 0.0027 mole, 1.0 mole eq.) and anhydrous dimethylformamide (10 ml) were placed in an oven dried, 50 ml three neck flask, equipped with a dropping funnel, inert gas inlet/outlet and a thermometer. Morpholine (0.26 gr, 0.0029 mole, 1.1 mole eq.) was added at 20-25°C. The reaction mixture was stirred for 1 hour at room temperature, and then concentrated to dryness under reduced pressure at 45-50°C to yield a residue. Hexane (25 ml) was added and stirred for 10 minutes to precipitate the desired regio-isomeric mixture product as a off-white solid (1.12 gr, Yield: 91 %, Combined purity of both isomers by HPLC and mass spectrometry: >99%).

Synthesis of Adenosine, 5'-*S*-methyl-5'-thio-, 2',3'-diacetate (Compound #68):

Scheme:

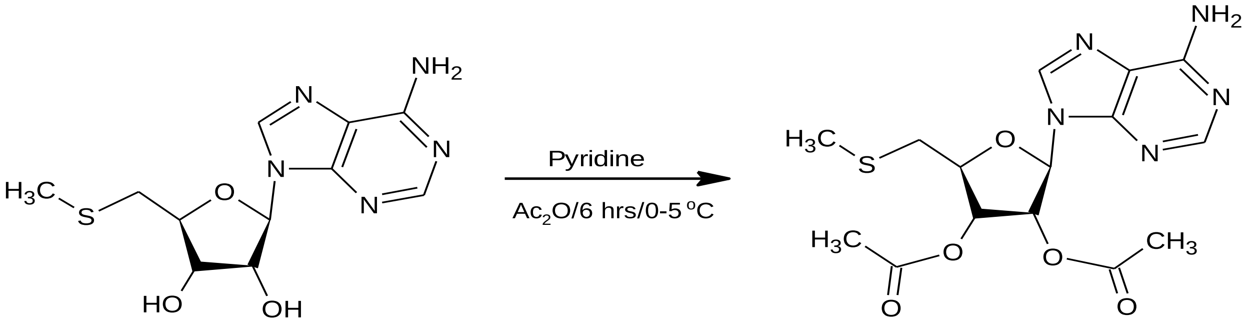

Synthetic procedure: 5'-*S*-methyl-5'-thio- Adenosine (1.0 gr, 0.0033 mole, 1.0 mole eq.) and anhydrous pyridine (10 ml) were placed in an oven dried, 50 ml three neck flask, equipped with a dropping funnel, inert gas inlet/outlet and a thermometer. The reaction set was placed in an ice/salt bath and agitation was initiated. When the temperature of the solution dropped to 0°C, acetic anhydride (10 ml, 0.105 mole, 31.8 mole eq.) was added drop-wise for 15 minutes and the temperature of the reaction mixture was maintained below 5°C during acetic anhydride addition. The reaction mixture was stirred for 6 hours at 5-10°C. The excess acetic anhydride was quenched by adding ice-cold water (100 ml), and then pH adjusted to 7 by adding 10 wt% $NaHCO_3$ aqueous solution. The aqueous mixture was extracted with ethyl acetate (2 x 100 ml). The combined ethyl acetate extracts were dried over anhydrous Na_2SO_4 (1gr) and filtered into a 250 ml round-bottomed flask. The filtrate was concentrated to dryness under reduced pressure at 35-40°C to give the crude product as a pale yellow syrupy liquid. Pure product was then obtained as off-white crystals (1.08 gr, Yield: 87 %, Purity by HPLC and mass spectrometry: >99%) by passing the yellow liquid through a silica gel column with a mixture of ethyl acetate and hexane (1:3 v/v).

Synthesis of Adenosine, 5'-*Se*-methyl-5'-seleno-, 2',3'-dipropionate (Compound #69):

Scheme:

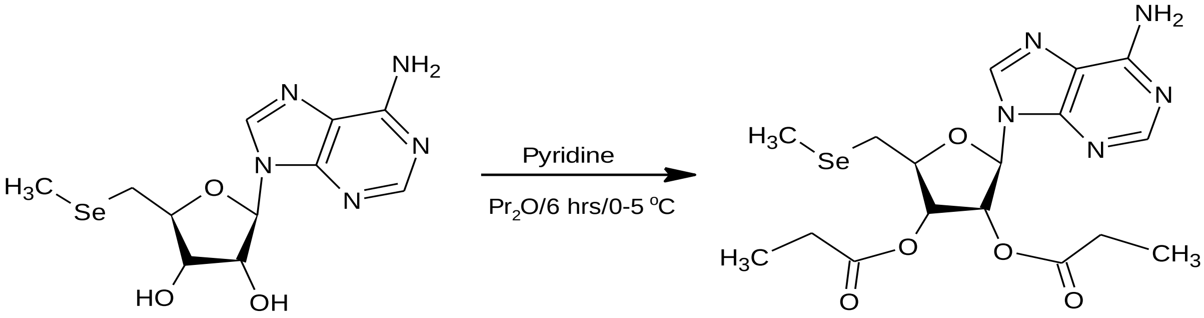

Synthetic procedure: 5'-*Se*-methyl-5'-seleno-Adenosine (1.0 gr, 0.0029 mole, 1.0 mole eq.) and anhydrous pyridine (10 ml) were placed in an oven dried, 50 ml three neck flask, equipped with a dropping funnel, inert gas inlet/outlet and a thermometer. The reaction set was placed in an ice/salt bath and agitation was initiated. When the temperature of the solution dropped to 0°C, propionic anhydride (10 ml, 0.078 mole, 27.0 mole eq.) was added drop-wise for 15 minutes and the temperature of the reaction mixture was maintained below 5°C during propionic anhydride addition. The reaction mixture was stirred for 6 hours at 0-5°C. The excess propionic anhydride was quenched by adding ice-cold water (100 ml), and then pH adjusted to 7 by adding 10 wt% $NaHCO_3$ aqueous solution. The aqueous mixture was extracted with ethyl acetate (2 x 100 ml). The combined ethyl acetate extracts were dried over anhydrous Na_2SO_4 (1 gr), filtered into a 250 ml round-bottomed flask. The filtrate was concentrated to dryness under reduced pressure at 35-40°C to give the crude product as a pale yellow syrupy liquid. The pure product was obtained as off-white crystals (1.18 gr, Yield: 89.3 %, Purity by HPLC and mass spectrometry: >99%) by passing the yellow liquid through a silica gel column with a mixture of ethyl acetate and hexanes (1:3 v/v).

Synthesis of Adenosine, 5'-*Se*-methyl-5'-seleno-, 2',3'-dibutanoate (Compound #70):

Scheme:

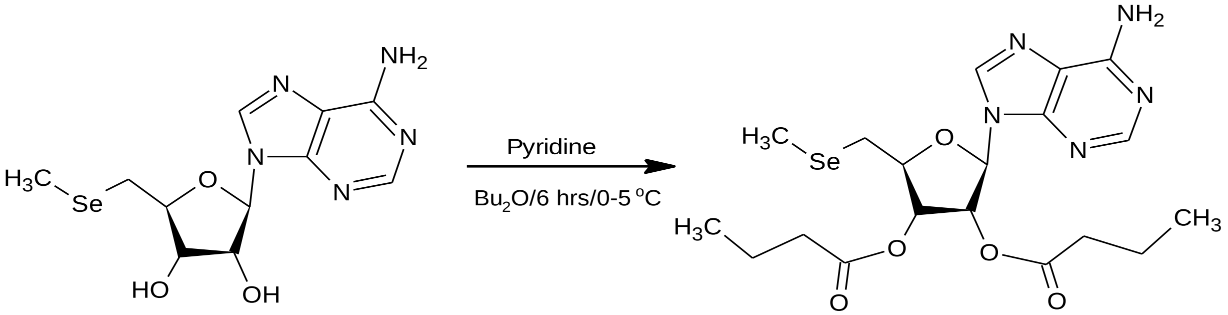

Synthetic procedure: 5'-*Se*-methyl-5'-seleno-Adenosine (1.0 gr, 0.0029 mole, 1.0 mole eq.) and anhydrous pyridine (10 ml) were placed in an oven dried, 50 ml three neck flask, equipped with a dropping funnel, inert gas inlet/outlet and a thermometer. The reaction set was placed in an ice/salt bath and agitation was initiated. When the temperature of the solution dropped to 0^oC, butyric anhydride (10 ml, 0.078 mole, 27.0 mole eq.) was added drop-wise for 15 minutes and the temperature of the reaction mixture was maintained below 5^oC during butyric anhydride addition. The reaction mixture was stirred for 6 hours at $0\text{-}5^oC$. The excess butyric anhydride was quenched by adding ice-cold water (100 ml), and then pH adjusted to 7 by adding 10 wt % $NaHCO_3$ aqueous solution. The aqueous mixture was extracted with ethyl acetate (2 x 100 ml). The combined ethyl acetate extracts were dried over anhydrous Na_2SO_4 (1 gr), filtered into a 250 ml round-bottomed flask. The filtrate was concentrated to dryness under reduced pressure at 35-40^oC to give the crude product as a pale yellow syrupy liquid. The pure product was obtained as off-white crystals (1.20 gr, Yield: 85.7 %, Purity by HPLC and mass spectrometry: >99%) by passing the yellow liquid through a silica gel column with a mixture of ethyl acetate and hexanes (1:3 v/v).
