# Supplementary material for: Non-peptidyl small molecule, adenosine, 5′-Se-methyl-5′-seleno-, 2′,3′-diacetate, activates insulin receptor and attenuates hyperglycemia in type 2 diabetic Leprdb/db mice
Source: Cell Mol Life Sci. 2019 Aug 5;77(8):1623–43. doi: 10.1007/s00018-019-03249-4 (PMC7162833; doi:10.1007/s00018-019-03249-4)
Supplement: Supplementary file 2 — Supplementary material 2 (PDF 108 kb) [file 18_2019_3249_MOESM2_ESM.pdf]

Title: Non-peptidyl small molecule, adenosine, 5'-Se-methyl-5'-seleno-, 2',3'-diacetate, activates insulin receptor and attenuates hyperglycemia in type 2 diabetic *Lepr*$^{db/db}$ mice

Journal: Cellular and Molecular Life Sciences

Authors: Zi-Jian Lan, Zhenmin Lei, Alexandros Yiannikouris, Thirupathi Reddy Yerramreddy, Xian Li, Hayley Kincaid, Katie Eastridge, Hannah Gadberry, Chloe Power, Rijin Xiao, Lei Lei, Olivia Seale, Karl Dawson and Ronan Power

Correspondence to: zlan@alltech.com and rpower@alltech.com

Supplementary Material-2

The list of Sigma's compounds screened in HepG2 cells.

Abbreviated name	Compound name	Compound structure	Molecular weight	Vendor
#57	Adenosine	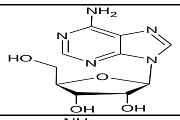	267.24	Sigma Cat#A4036
#59	2',5'-Dideoxy-adenosine	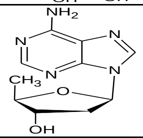	235.24	Sigma Cat#D7408
#60	5'-Deoxyadenosine	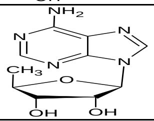	251.24	Sigma Cat#1771
#61	SQ 22,536	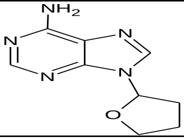	205.21	Sigma Cat#S153
#62	Adenine	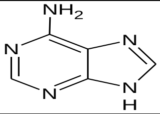	135.12	Sigma Cat#2786
#63	β-Nicotinamide adenine dinucleotide hydrate (NAD)	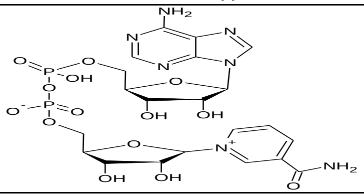	664.43	Sigma Cat#NAD 100-RO
#64	S-(5'-Adenosyl)-L-methionine iodide (SAM)	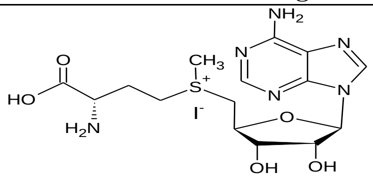	526.35	Sigma Cat#A4377
