# Supplementary material for: Non-peptidyl small molecule, adenosine, 5′-Se-methyl-5′-seleno-, 2′,3′-diacetate, activates insulin receptor and attenuates hyperglycemia in type 2 diabetic Leprdb/db mice
Source: Cell Mol Life Sci. 2019 Aug 5;77(8):1623–43. doi: 10.1007/s00018-019-03249-4 (PMC7162833; doi:10.1007/s00018-019-03249-4)
Supplement: Supplementary file 3 — Supplementary material 3 (PDF 41 kb) [file 18_2019_3249_MOESM3_ESM.pdf]

Title: Non-peptidyl small molecule, adenosine, 5'-Se-methyl-5'-seleno-, 2',3'-diacetate, activates insulin receptor and attenuates hyperglycemia in type 2 diabetic *Lepr*$^{db/db}$ mice

Journal: Cellular and Molecular Life Sciences

Authors: Zi-Jian Lan, Zhenmin Lei, Alexandros Yiannikouris, Thirupathi Reddy Yerramreddy, Xian Li, Hayley Kincaid, Katie Eastridge, Hannah Gadberry, Chloe Power, Rijin Xiao, Lei Lei, Olivia Seale, Karl Dawson and Ronan Power

Correspondence to: zlan@alltech.com and rpower@alltech.com

Supplementary Material-3

Mass spectrometry analysis of NPC43 in the *in vitro* binding assay

A linear calibration curve (non-forced through the origin) was created by preparing serial dilutions of NPC43 (233.5, 116.7, 58.4, 29.2, 14.6, 7.3, 3.7, 1.8 and 0.9 nM). All supernatant samples (after removal of Dynabeads) were diluted up to 3 times with 5:95, methanol:H2O, 0.1% formic acid solution to reach the calibration range.

NPC43 was analyzed using a UPLC-ESI-VionIMS-QTOF instrument equipped with a RP-C8 analytical column (2.1 mm × 100 mm, Waters Corp) maintained at 30.0°C. Ten microliters of samples, maintained at ~5°C for the entire analysis, were injected in the system. A binary mobile phase consisting of MS-grade Ultrapure water with 0.1% formic acid (eluent A) and methanol containing 0.1% formic acid (eluent B) was used. The flow rate was set to 0.40 mL/min and the total analysis time was 12 min using a gradient elution. All samples were injected in random order. The IMS-QTOF instrument was fitted with an Electro-Spray Ionization interface (ESI) operated in positive mode; NPC43 forming primarily a positive protonated ion [M+H]+ (^{80}Se = 430.061 m/z). Source was operated at a capillary voltage of 3.00 kV and a temperature of 120°C. N_2 desolvation gas temperature, gas flow and cone gas flow were set up at 650°C, 800L/hr and 50L/hr, respectively.

The identity of NPC43 was confirmed by high mass accuracy measurement (monoisotopic mass of 429.0551 g.mol^{-1}); according to the isotopic profile of selenium; according to the specific retention time of NPC43 (7.3 min).

Quantification was performed by evaluating the area under the curve (elution peak) for NPC43 compared to the signal response of a 9-standard concentration calibration curve, defined by a linear regression. A calibration regression curve was constructed with a coefficient of regression of r^2 = 0.997046.
