# Supplementary material for: Non-peptidyl small molecule, adenosine, 5′-Se-methyl-5′-seleno-, 2′,3′-diacetate, activates insulin receptor and attenuates hyperglycemia in type 2 diabetic Leprdb/db mice
Source: Cell Mol Life Sci. 2019 Aug 5;77(8):1623–43. doi: 10.1007/s00018-019-03249-4 (PMC7162833; doi:10.1007/s00018-019-03249-4)
Supplement: Supplementary file 4 — Supplementary material 4 (PDF 158 kb) [file 18_2019_3249_MOESM4_ESM.pdf]

Title: Non-peptidyl small molecule, adenosine, 5'-Se-methyl-5'-seleno-, 2',3'-diacetate, activates insulin receptor and attenuates hyperglycemia in type 2 diabetic *Lepr*$^{db/db}$ mice

Journal: Cellular and Molecular Life Sciences

Authors: Zi-Jian Lan, Zhenmin Lei, Alexandros Yiannikouris, Thirupathi Reddy Yerramreddy, Xian Li, Hayley Kincaid, Katie Eastridge, Hannah Gadberry, Chloe Power, Rijin Xiao, Lei Lei, Olivia Seale, Karl Dawson and Ronan Power

Correspondence to: zlan@alltech.com and rpower@alltech.com

Supplementary Material-4

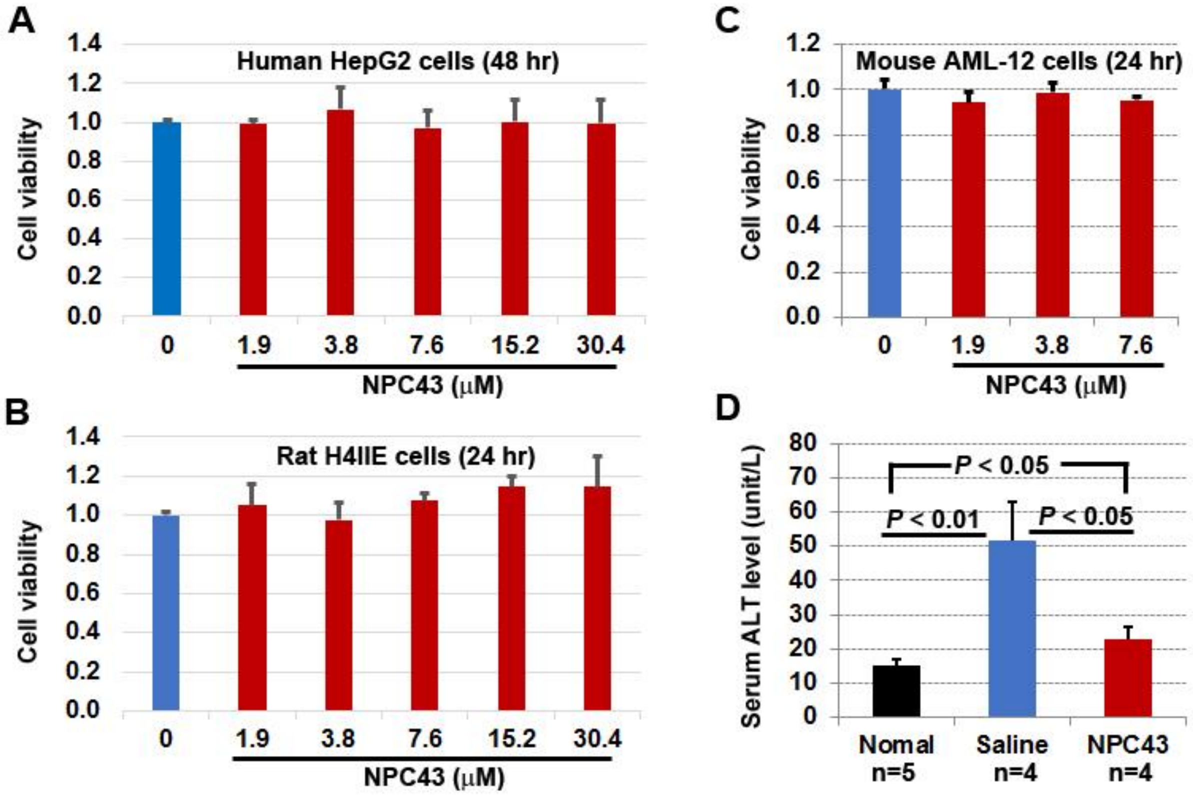

Effects of NPC43 on (A-C) cultured liver cell viability and (D) serum ALT levels in *Lepr*$^{db/db}$ mice. (**A-C**) No toxic effects of NPC43 on cell viability in cultured human, rat and mouse liver cells. Equal numbers of (**A**) HepG2, (**B**) H4IIE cells or (**C**) AML-12 cells were seeded on 96-well

plates, cultured overnight, treated with NPC43 solvent (0.24% DMSO, referred to as zero control) or NPC43 (1.9-30.4 μM) in serum-free media at 37°C for 24 hr (H4IIE and AML-12 cells) or 48 hr (HepG2 cell only), and then subjected to cell viability analysis. Data are presented as mean ± SD of four replicates per group and *P* value was higher than 0.05 (all NPC43-treated groups vs. the zero control group, *Student's t-test*). **(D)** ALT levels in the sera of *Lepr$^{db/db}$* mice after chronic treatment with NPC43. *Lepr$^{db/db}$* mice were intraperitoneally injected with 0.2% (v/v) DMSO/physiological saline or NPC43 (0.136 mg/kg BW) daily for 52 days. Then, sera from these DMSO/saline- and NPC43-treated *Lepr$^{db/db}$* mice, as well as age-matched (3-month-old) wild-type C57 mice (without treatments, referred to as normal group), were subjected to ALT analysis. Data are presented as Mean ± SEM of indicated number of mice per group, and *Student's t-test* was performed to obtain the *P* value between indicated two groups.
