# Supplementary material for: Non-peptidyl small molecule, adenosine, 5′-Se-methyl-5′-seleno-, 2′,3′-diacetate, activates insulin receptor and attenuates hyperglycemia in type 2 diabetic Leprdb/db mice
Source: Cell Mol Life Sci. 2019 Aug 5;77(8):1623–43. doi: 10.1007/s00018-019-03249-4 (PMC7162833; doi:10.1007/s00018-019-03249-4)
Supplement: Supplementary file 5 — Supplementary material 5 (PDF 166 kb) [file 18_2019_3249_MOESM5_ESM.pdf]

Title: Non-peptidyl small molecule, adenosine, 5'-Se-methyl-5'-seleno-, 2',3'-diacetate, activates insulin receptor and attenuates hyperglycemia in type 2 diabetic *Lepr*$^{db/db}$ mice

Journal: Cellular and Molecular Life Sciences

Authors: Zi-Jian Lan, Zhenmin Lei, Alexandros Yiannikouris, Thirupathi Reddy Yerramreddy, Xian Li, Hayley Kincaid, Katie Eastridge, Hannah Gadberry, Chloe Power, Rijin Xiao, Lei Lei, Olivia Seale, Karl Dawson and Ronan Power

Correspondence to: zlan@alltech.com and rpower@alltech.com

Supplementary Material-5

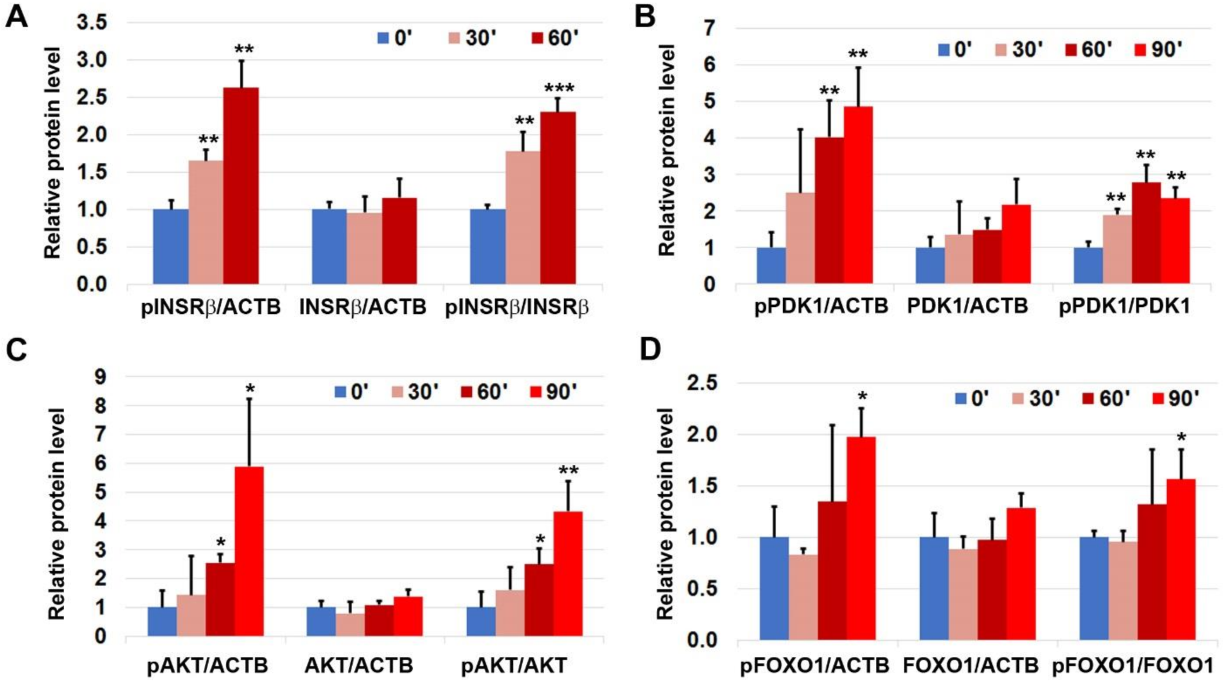

Quantitative changes in protein expression of (A) pINSRβ/INSRβ, (B) pPDK1/PDK1, (C) pAKT/AKT and (D) pFOXO1/FOXO1 in human liver HepG2 cells after treatment with NPC43 (7.6 μM) for 30, 60 or 90 minutes, as determined by Western blot analysis shown in Fig. 4A. Densities of protein bands shown in Western blots (Fig. 4A) were determined using NIH Image J software. Data are presented as mean ± SD of three replicates per group. * $P < 0.05$, ** $P < 0.01$, *** $P < 0.001$, when compared to the protein level at 0 min time point (right before compound treatment) (*Student's t-test*).
