# Supplementary material for: Non-peptidyl small molecule, adenosine, 5′-Se-methyl-5′-seleno-, 2′,3′-diacetate, activates insulin receptor and attenuates hyperglycemia in type 2 diabetic Leprdb/db mice
Source: Cell Mol Life Sci. 2019 Aug 5;77(8):1623–43. doi: 10.1007/s00018-019-03249-4 (PMC7162833; doi:10.1007/s00018-019-03249-4)
Supplement: Supplementary file 7 — Supplementary material 7 (PDF 242 kb) [file 18_2019_3249_MOESM7_ESM.pdf]

Title: Non-peptidyl small molecule, adenosine, 5'-Se-methyl-5'-seleno-, 2',3'-diacetate, activates insulin receptor and attenuates hyperglycemia in type 2 diabetic *Lepr*$^{db/db}$ mice

Journal: Cellular and Molecular Life Sciences

Authors: Zi-Jian Lan, Zhenmin Lei, Alexandros Yiannikouris, Thirupathi Reddy Yerramreddy, Xian Li, Hayley Kincaid, Katie Eastridge, Hannah Gadberry, Chloe Power, Rijin Xiao, Lei Lei, Olivia Seale, Karl Dawson and Ronan Power

Correspondence to: zlan@alltech.com and rpower@alltech.com

Supplementary Material-7:

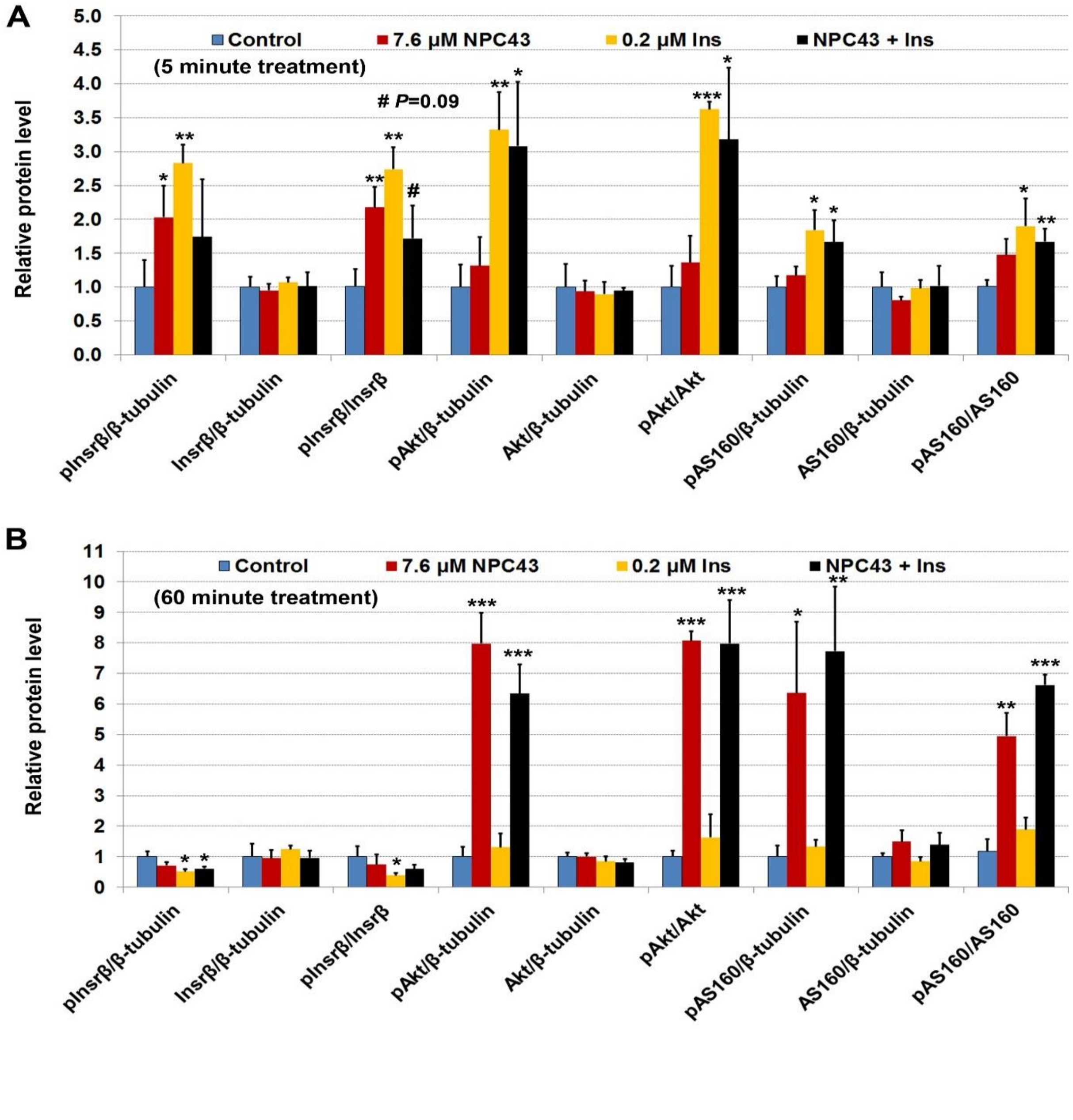

Quantitative changes in protein expression of pInsrβ Y1146 and its downstream signaling molecules, Akt and AS160, in differentiated C2C12 (skeletal muscle) cells after treatment with NPC43 (7.6 μM), insulin (Ins, 0.2 μM) or both for (A) 5 and (B) 60 minutes, as determined by Western blot analysis shown in Fig. 6A. Densities of protein bands in Western blots (Fig. 6A) were determined using NIH Image J software. Data are presented as mean ± SD of triplicates per group. *$P < 0.05$, **$P < 0.01$, ***$P < 0.001$, vs. its control group (without NPC43 treatment group, *Student's t-test*).
