# Supplementary material for: Non-peptidyl small molecule, adenosine, 5′-Se-methyl-5′-seleno-, 2′,3′-diacetate, activates insulin receptor and attenuates hyperglycemia in type 2 diabetic Leprdb/db mice
Source: Cell Mol Life Sci. 2019 Aug 5;77(8):1623–43. doi: 10.1007/s00018-019-03249-4 (PMC7162833; doi:10.1007/s00018-019-03249-4)
Supplement: Supplementary file 8 — Supplementary material 8 (PDF 137 kb) [file 18_2019_3249_MOESM8_ESM.pdf]

Title: Non-peptidyl small molecule, adenosine, 5'-Se-methyl-5'-seleno-, 2',3'-diacetate, activates insulin receptor and attenuates hyperglycemia in type 2 diabetic *Lepr$^{db/db}$* mice

Journal: Cellular and Molecular Life Sciences

Authors: Zi-Jian Lan, Zhenmin Lei, Alexandros Yiannikouris, Thirupathi Reddy Yerramreddy, Xian Li, Hayley Kincaid, Katie Eastridge, Hannah Gadberry, Chloe Power, Rijin Xiao, Lei Lei, Olivia Seale, Karl Dawson and Ronan Power

Correspondence to: zlan@alltech.com and rpower@alltech.com

Supplementary Material-8:

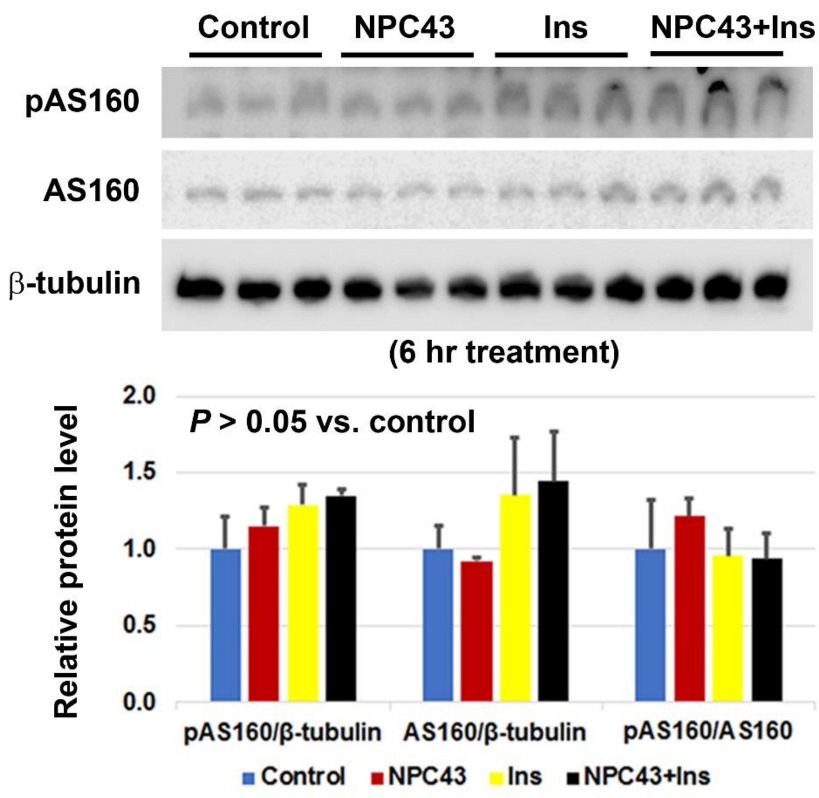

No significant increase in AS160 phosphorylation in differentiated C2C12 cells after treatment with NPC43, insulin or both NPC43 and insulin for 6 hr. Completely differentiated C2C12 cells were serum-starved overnight, treated without (Control) or with NPC43 (7.6 μM), insulin (0.2 μM) or both in serum-free DMEM media at 37°C for 6 hr, and then subjected to Western blot analysis (using 8 μg protein, triplicates/group). Densities of those protein bands shown in Western blots were determined using NIH Image J software, and then normalized by β-tubulin or total AS160 protein level. Data are presented as mean ± SD of triplicates per group. There was no significant ($P > 0.05$) increase in pAS160 or AS160 protein levels as well as the ratios of pAS160 to AS160 in NPC43-treated groups when compared to the control group (*Student's t-test*).
