# Supplementary material for: Non-peptidyl small molecule, adenosine, 5′-Se-methyl-5′-seleno-, 2′,3′-diacetate, activates insulin receptor and attenuates hyperglycemia in type 2 diabetic Leprdb/db mice
Source: Cell Mol Life Sci. 2019 Aug 5;77(8):1623–43. doi: 10.1007/s00018-019-03249-4 (PMC7162833; doi:10.1007/s00018-019-03249-4)
Supplement: Supplementary file 9 — Supplementary material 9 (PDF 162 kb) [file 18_2019_3249_MOESM9_ESM.pdf]

Title: Non-peptidyl small molecule, adenosine, 5'-Se-methyl-5'-seleno-, 2',3'-diacetate, activates insulin receptor and attenuates hyperglycemia in type 2 diabetic *Lepr*$^{db/db}$ mice

Journal: Cellular and Molecular Life Sciences

Authors: Zi-Jian Lan, Zhenmin Lei, Alexandros Yiannikouris, Thirupathi Reddy Yerramreddy, Xian Li, Hayley Kincaid, Katie Eastridge, Hannah Gadberry, Chloe Power, Rijin Xiao, Lei Lei, Olivia Seale, Karl Dawson and Ronan Power

Correspondence to: zlan@alltech.com and rpower@alltech.com

Supplementary Material-9

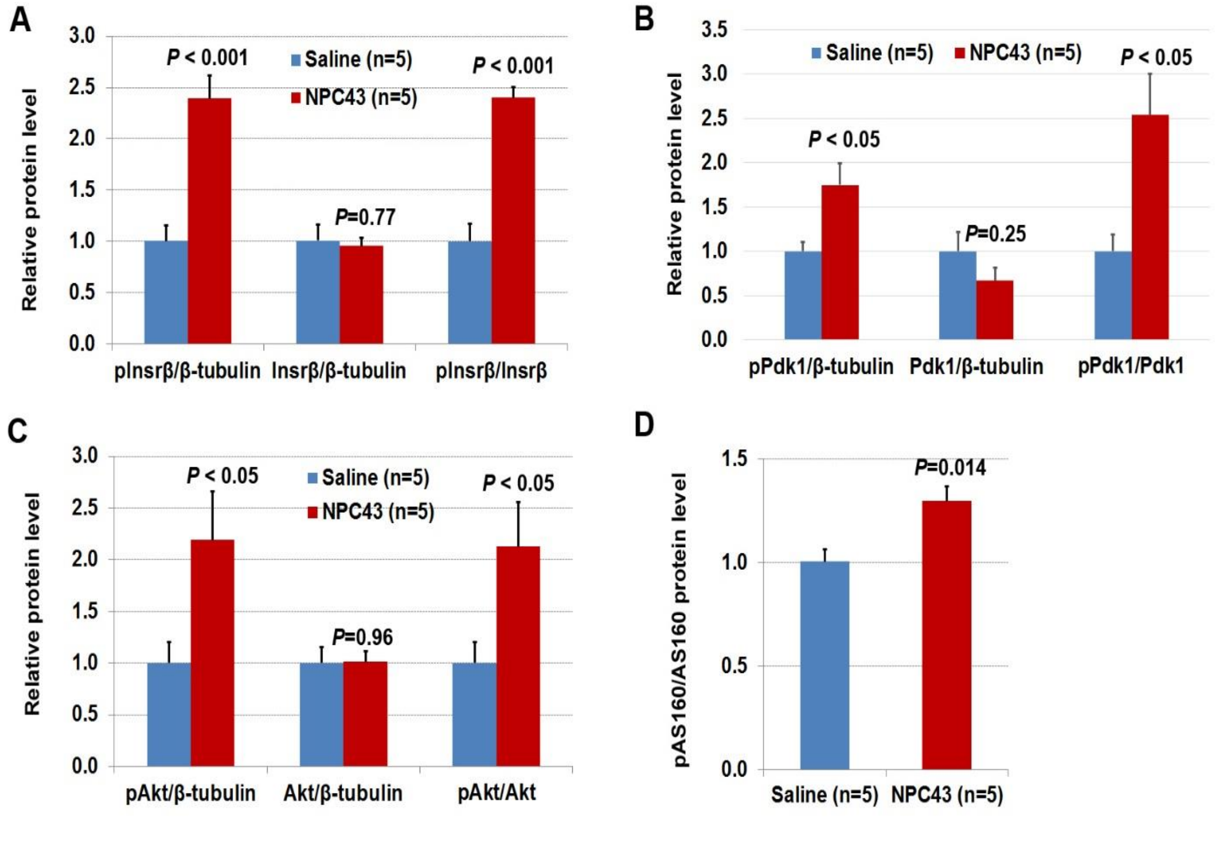

Quantitative changes in protein expression of (A) pInsrβ Y1146/Insrβ, (B) pPdk1 S241/Pdk1, (C) pAkt T308/Akt, and (D) pAS160 S588/AS160 in the skeletal muscle of *Lepr*$^{db/db}$ mice after chronic i.p. treatment with NPC43, as determined by Western blot analysis and/or immunoprecipitation shown in Fig. 6B. Protein band densities in Western blots in Fig. 6B were determined using NIH Image J software, and data are presented as mean ± SEM of 5 mice per group. *P* value (the NPC43-treated group vs. the control saline group) was determined by performing *Student's t-test.*
