# Supplementary material for: Non-peptidyl small molecule, adenosine, 5′-Se-methyl-5′-seleno-, 2′,3′-diacetate, activates insulin receptor and attenuates hyperglycemia in type 2 diabetic Leprdb/db mice
Source: Cell Mol Life Sci. 2019 Aug 5;77(8):1623–43. doi: 10.1007/s00018-019-03249-4 (PMC7162833; doi:10.1007/s00018-019-03249-4)
Supplement: Supplementary file 10 — Supplementary material 10 (PDF 282 kb) [file 18_2019_3249_MOESM10_ESM.pdf]

Title: Non-peptidyl small molecule, adenosine, 5'-Se-methyl-5'-seleno-, 2',3'-diacetate, activates insulin receptor and attenuates hyperglycemia in type 2 diabetic *Lepr*$^{db/db}$ mice

Journal: Cellular and Molecular Life Sciences

Authors: Zi-Jian Lan, Zhenmin Lei, Alexandros Yiannikouris, Thirupathi Reddy Yerramreddy, Xian Li, Hayley Kincaid, Katie Eastridge, Hannah Gadberry, Chloe Power, Rijin Xiao, Lei Lei, Olivia Seale, Karl Dawson and Ronan Power

Correspondence to: zlan@alltech.com and rpower@alltech.com

Supplementary Material-10

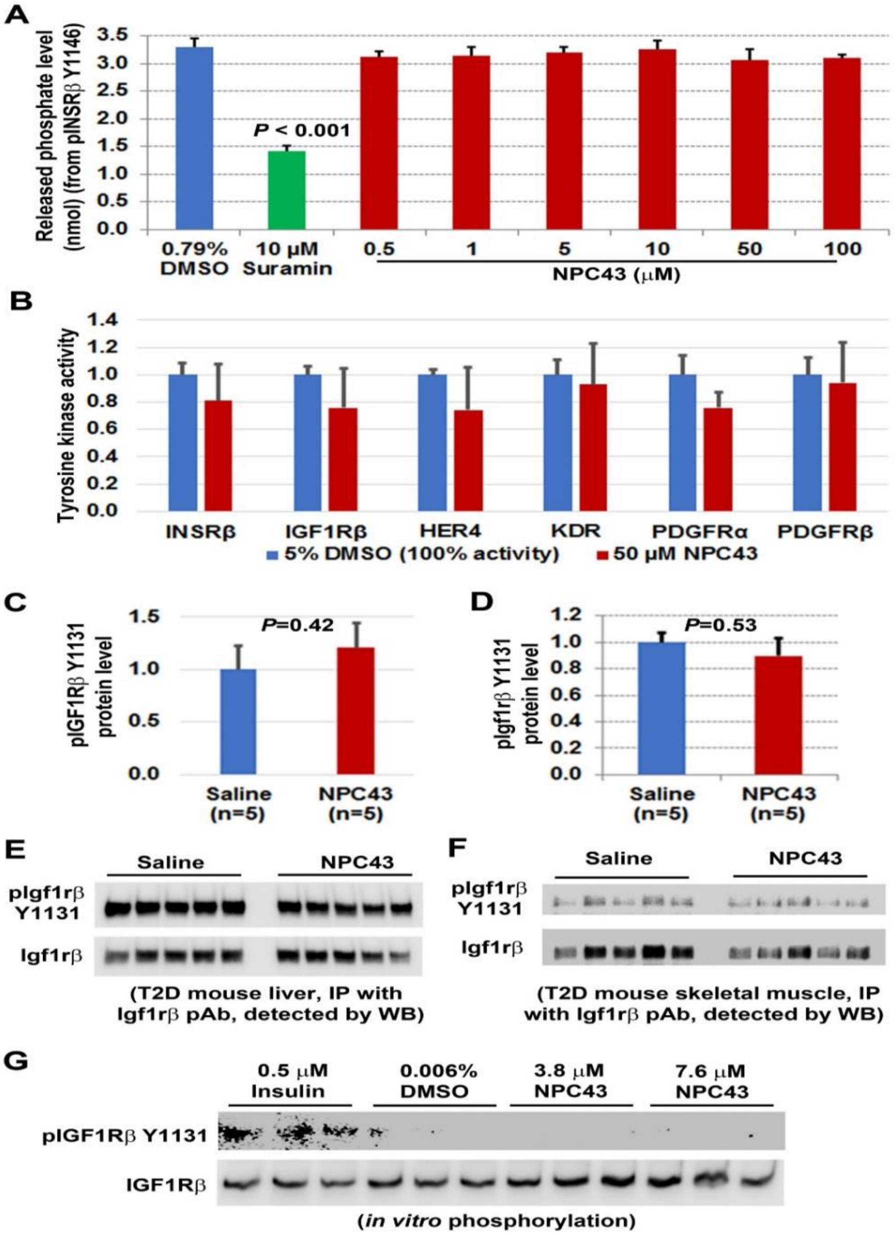

Effects of NPC43 on (A-B) the activities of intracellular PTP1B and several major tyrosine kinases *in vitro*, (C-E) the activation of endogenous Igf1r in liver and skeletal muscle of *Lepr*$^{db/db}$ mice, and (G) the activation of IGF1R protein in a cell-free *in vitro* phosphorylation system. **(A)** No effects of NPC43 on the PTP1B activity *in vitro*. Recombinant PTP1B (2.5 ng) and its substrate (75 μM, INSRβ amino acid residues 1142-1153, pY-1146) were incubated with 0.79% (v/v) DMSO (the NPC43 solvent), suramin (a positive inhibitor, 10 μM) or 1-100 μM NPC43 and subjected to *in vitro* PTP1B assays to determine the released phosphate levels. Mean ± SD of triplicates per group. *P* values (vs 0.79%DMSO group) in all NPC43-treated groups was higher than 0.05 (*Student's t-test*). **(B)** No enhanced tyrosine kinase activity of INSRβ, IGF1Rβ, HER4, KDR, PDGFRα or PDGFRβ *in vitro* after NPC43 treatment, as determined by *in vitro* receptor tyrosine kinase assays. The NPC43 solvent [5% (v/v) DMSO in PBS buffer) was included as a positive control (referred to 100% kinase activity). Data are presented as mean ± SD of triplicates per group. *P* values in all NPC43-treated groups were higher than 0.05 (vs. the 5% DMSO group, *Student's t-test*). **(C-F)** No significant increase in protein levels of pIgf1rβ at Y1131 in the **(C, E)** liver and **(D, F)** gastrocnemius of *Lepr*$^{db/db}$ mice after chronic i.p. treatment with NPC43, as determined by **(C-D)** ELISA and **(E-F)** co-immunoprecipitation analysis. Male *Lepr*$^{db/db}$ mice at postnatal day 38 were i.p. injected daily with 0.2% (v/v) DMSO/physiological saline (referred to as the saline group) or NPC43 (0.136 mg/kg BW) for 52 days. Liver protein extracts (50 μg protein) or skeletal muscle protein extracts (150 μg protein) from five saline- or NPC43-treated mice were subjected to **(C-D)** ELISA analysis of pIgf1rβY1131**.** Data in **C-D** are presented as mean ± SEM, and *P* values between saline control and NPC43-treated groups were determined by performing *Student's t-test.* In **(E-F)**, liver or skeletal muscle protein extracts (600 μg) from five saline- or NPC43-treated mice were immunoprecipitated with a specific Igf1r antibody followed by Western blot analysis of pIgf1rβ at Y1131 and total Igf1rβ in these immunoprecipitated samples. **(G)** No activation of IGF1R protein by NPC43 in the cell-free *in vitro* phosphorylation system. Equal amounts of purified IGF1R protein (400 ng, triplicates per group) were incubated with 0.5 μM insulin, 0.006% (v/v) DMSO or NPC43 (3.8 or 7.6 μM) and subjected to *in vitro* phosphorylation analysis. Activated IGF1R (i.e. IGF1Rβ Y1131) and total IGF1Rβ protein in the *in vitro* phosphorylation reactions were detected by Western blot analysis. Experiments were repeated three times. Note the activation of IGF1R by insulin, but not by NPC43 at the tested doses.
