# Supplementary material for: Non-peptidyl small molecule, adenosine, 5′-Se-methyl-5′-seleno-, 2′,3′-diacetate, activates insulin receptor and attenuates hyperglycemia in type 2 diabetic Leprdb/db mice
Source: Cell Mol Life Sci. 2019 Aug 5;77(8):1623–43. doi: 10.1007/s00018-019-03249-4 (PMC7162833; doi:10.1007/s00018-019-03249-4)
Supplement: Supplementary file 11 — Supplementary material 11 (PDF 197 kb) [file 18_2019_3249_MOESM11_ESM.pdf]

Title: Non-peptidyl small molecule, adenosine, 5'-Se-methyl-5'-seleno-, 2',3'-diacetate, activates insulin receptor and attenuates hyperglycemia in type 2 diabetic *Lepr*$^{db/db}$ mice

Journal: Cellular and Molecular Life Sciences

Authors: Zi-Jian Lan, Zhenmin Lei, Alexandros Yiannikouris, Thirupathi Reddy Yerramreddy, Xian Li, Hayley Kincaid, Katie Eastridge, Hannah Gadberry, Chloe Power, Rijin Xiao, Lei Lei, Olivia Seale, Karl Dawson and Ronan Power

Correspondence to: zlan@alltech.com and rpower@alltech.com

Supplementary Material-11

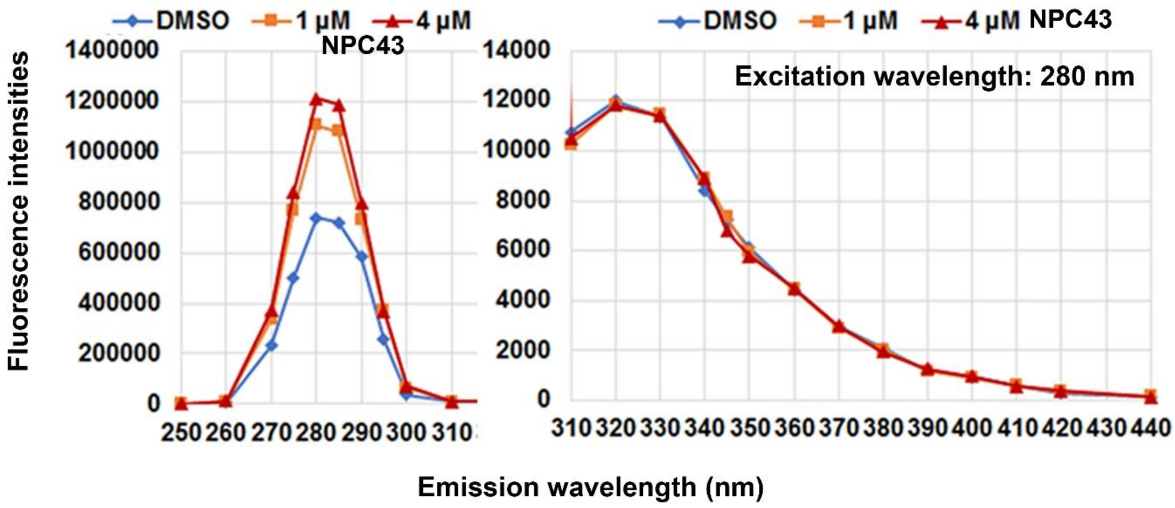

NPC43 alone had intrinsic fluorescence at Em 270-300 nm/Ex 280 nm (which overlaps the Phe/Tyr fluorescence of recombinant INSRα protein) but not at Em 310-440 nm/Ex 280 nm. The fluorescence spectra of DMSO (0.006%, v/v, the NPC43 solvent) and NPC43 (1 µM or 4 µM) in plain 1X PBS buffer at the emission wavelengths from 250 to 440 nm with an excitation wavelength of 280 nm were recorded by a fluorometer. Note the increased fluorescence in NPC43 solutions at Em 270-300 nm/Ex 280 nm but no change in fluorescence at Em 310-440 nm/Ex 280 nm (vs. the DMSO solution).
