# Supplementary material for: Non-peptidyl small molecule, adenosine, 5′-Se-methyl-5′-seleno-, 2′,3′-diacetate, activates insulin receptor and attenuates hyperglycemia in type 2 diabetic Leprdb/db mice
Source: Cell Mol Life Sci. 2019 Aug 5;77(8):1623–43. doi: 10.1007/s00018-019-03249-4 (PMC7162833; doi:10.1007/s00018-019-03249-4)
Supplement: Supplementary file 12 — Supplementary material 12 (PDF 162 kb) [file 18_2019_3249_MOESM12_ESM.pdf]

Title: Non-peptidyl small molecule, adenosine, 5'-Se-methyl-5'-seleno-, 2',3'-diacetate, activates insulin receptor and attenuates hyperglycemia in type 2 diabetic *Lepr*$^{db/db}$ mice

Journal: Cellular and Molecular Life Sciences

Authors: Zi-Jian Lan, Zhenmin Lei, Alexandros Yiannikouris, Thirupathi Reddy Yerramreddy, Xian Li, Hayley Kincaid, Katie Eastridge, Hannah Gadberry, Chloe Power, Rijin Xiao, Lei Lei, Olivia Seale, Karl Dawson and Ronan Power

Correspondence to: zlan@alltech.com and rpower@alltech.com

Supplementary Material-12

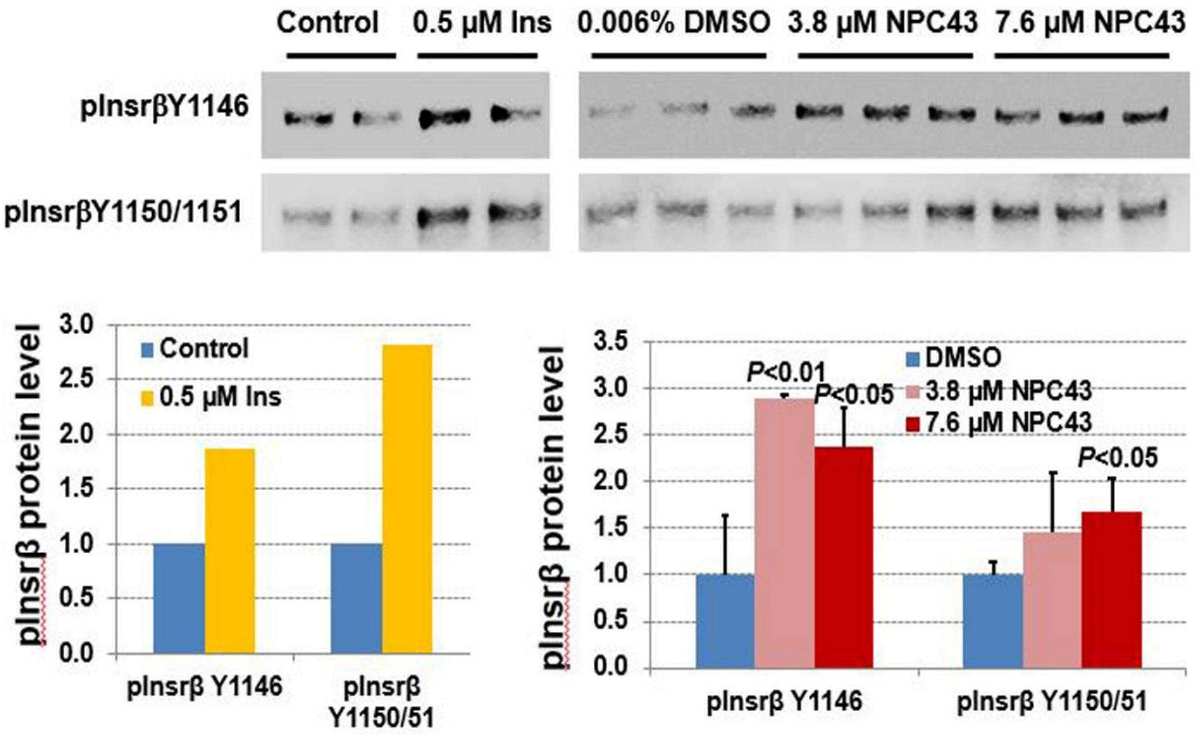

Activation of rat liver Insr protein by 0.5 μM insulin and 3.8-7.6 μM NPC43, as determined by cell-free *in vitro* phosphorylation assays. Equal amounts of purified rat liver Insr protein (final concentration: 1.63 μM) were incubated without (control, duplicates) or with 0.5 μM insulin (duplicates), 0.006% (v/v) DMSO (the NPC43 solvent, triplicates), or NPC43 (3.8 or 7.6 μM, triplicates/group) and then subjected to *in vitro* phosphorylation assays in the presence of ATP. Activated Insr (i.e. pInsrβ Y1146 and pInsrβ Y1150/1151) proteins in each sample was detected by Western blot analysis using its specific antibody. Protein band densities in Western blots were obtained using NIH Image J software and shown in bar graphs. In the left bar graph, data are presented as mean of duplicates per group. In the right bar graph, data are presented as Mean ± SD of triplicates per group and *P* value (the NPC43-treated groups vs. 0.006% DMSO group) was determined by performing *Student's t-test*.
